# Changes in the miRNA-mRNA Regulatory Network Precede Motor Symptoms in a Mouse Model of Multiple System Atrophy: Clinical Implications

**DOI:** 10.1371/journal.pone.0150705

**Published:** 2016-03-10

**Authors:** Simon Schafferer, Rimpi Khurana, Violetta Refolo, Serena Venezia, Edith Sturm, Paolo Piatti, Clara Hechenberger, Hubert Hackl, Roman Kessler, Michaela Willi, Ronald Gstir, Anne Krogsdam, Alexandra Lusser, Werner Poewe, Gregor K. Wenning, Alexander Hüttenhofer, Nadia Stefanova

**Affiliations:** 1 Division of Genomics and RNomics, Biocenter, Medical University of Innsbruck, Innrain 80–82, 6020 Innsbruck, Austria; 2 Division of Neurobiology, Department of Neurology, Medical University of Innsbruck, Innrain 66/G2, 6020 Innsbruck, Austria; 3 Division of Molecular Biology, Biocenter, Medical University of Innsbruck, Innrain 80–82, 6020 Innsbruck, Austria; 4 Division of Bioinformatics, Biocenter, Medical University of Innsbruck, Innrain 80–82, 6020 Innsbruck, Austria; 5 Department of Neurology, Medical University of Innsbruck, Anichstr. 35, 6020 Innsbruck, Austria; NIH, UNITED STATES

## Abstract

Multiple system atrophy (MSA) is a fatal rapidly progressive α-synucleinopathy, characterized by α-synuclein accumulation in oligodendrocytes. It is accepted that the pathological α-synuclein accumulation in the brain of MSA patients plays a leading role in the disease process, but little is known about the events in the early stages of the disease. In this study we aimed to define potential roles of the miRNA-mRNA regulatory network in the early pre-motor stages of the disease, i.e., downstream of α-synuclein accumulation in oligodendroglia, as assessed in a transgenic mouse model of MSA. We investigated the expression patterns of miRNAs and their mRNA targets in substantia nigra (SN) and striatum, two brain regions that undergo neurodegeneration at a later stage in the MSA model, by microarray and RNA-seq analysis, respectively. Analysis was performed at a time point when α-synuclein accumulation was already present in oligodendrocytes at neuropathological examination, but no neuronal loss nor deficits of motor function had yet occurred. Our data provide a first evidence for the leading role of gene dysregulation associated with deficits in immune and inflammatory responses in the very early, non-symptomatic disease stages of MSA. While dysfunctional homeostasis and oxidative stress were prominent in SN in the early stages of MSA, in striatum differential gene expression in the non-symptomatic phase was linked to oligodendroglial dysfunction, disturbed protein handling, lipid metabolism, transmembrane transport and altered cell death control, respectively. A large number of putative miRNA-mRNAs interaction partners were identified in relation to the control of these processes in the MSA model. Our results support the role of early changes in the miRNA-mRNA regulatory network in the pathogenesis of MSA preceding the clinical onset of the disease. The findings thus contribute to understanding the disease process and are likely to pave the way towards identifying disease biomarkers for early diagnosis of MSA.

## Introduction

Multiple system atrophy (MSA) is a fatal, late onset, sporadic neurodegenerative disorder, which is characterized by a combination of non-motor and motor symptoms with rapid progression resulting in disability and death shortly after clinical diagnosis [1]. The neuropathology encompasses degeneration of autonomic centers as well as striatonigral degeneration (SND) and olivopontocerebellar atrophy (OPCA) that underlie respectively the Parkinson syndrome and the ataxia in MSA [2]. The major hallmark of the disease is the widespread occurrence of α-synuclein positive cytoplasmic inclusions in oligodendrocytes called glial cytoplasmic inclusions (GCIs) [3–5]. It is currently accepted that α-synuclein plays a major role in the pathogenesis of MSA [6]. Recent studies indicate that increased expression of α-synuclein in MSA oligodendroglia [7;8] may trigger the pathological aggregation of the protein in these cells and the following neurodegenerative events that bring forward selective neuronal loss resulting in the clinical symptoms of MSA. Little is known, however, about the early events in the disease cascade before its clinical onset due to the difficulties to address this question in patients because early diagnostic markers for MSA are lacking at present.

Several transgenic models based on the targeted overexpression of human α-synuclein in oligodendroglia have been developed by applying specific promoters, i. e. the 2,' 3'-cyclic nucleotide 3'-phosphodiesterase (CNP), the myelin basic protein (MBP) promoter or the proteolipid protein (PLP) promoter (for detailed comparison of the different transgenic MSA models see [9]). The transgenic mouse model of MSA, based on targeted overexpression of human α-synuclein in oligodendrocytes under the PLP promoter [10] recapitulates many of the features observed in MSA-like oligodendroglial α-synucleinopathy. In this transgenic model oligodendroglial α-synuclein pathology leads to slowly progressive motor deficits linked to delayed neuronal loss in the substantia nigra (SN) and striatum [11–13]. Therefore, the PLP-α-synuclein transgenic mouse is useful to address the early steps of MSA-like SND in its pre-motor phase.

Recent studies in MSA post-mortem brain tissue identified changes in the transcriptome expression profile [14]. Furthermore, possible dysregulation of microRNA (miRNA) expression has been reported at the end-stage of the disease [15]. While mRNAs are translated into proteins, non-coding RNAs (ncRNAs), in particular miRNAs, have been shown to regulate the expression of mRNAs by targeting their 5’- or 3’-untranslated regions (UTRs), thereby regulating their translation and/or mRNA stability. Hence, the interplay between miRNAs and mRNAs forms an intricate regulatory network and fine-tunes gene expression. MiRNAs are involved in numerous biological functions, including regulation of development and differentiation, apoptosis, or maintenance of cell integrity [16;17]. They have also been implicated in several CNS disorders, such as Alzheimer’s disease (AD), Parkinson’s disease (PD), or Huntington’s disease (HD), respectively [18–21]. In summary, the existing data suggest that miRNA-mRNA regulatory networks play a role in the pathogenesis of neurodegenerative disorders and we therefore hypothesize that they may have a role in MSA as well. In particular, early changes may be causal and may contribute to the pathogenesis of MSA, while late changes may represent a consequence of the already established neuronal and glial damage.

Hence, to get insights into the very early pathogenic mechanisms of neurodegeneration linked to oligodendroglial α-synuclein accumulation, in this study we investigated the miRNA-mRNA regulatory network in SN and striatum of MSA transgenic mice in a pre-motor stage of neurodegeneration.

## Material and Methods

### Animals

The generation and characterization of the PLP-α-synuclein mice in C57Bl/6 background was previously described [10]. Homozygous transgenic PLP-α-synuclein mice (further also termed MSA transgenic mice) obtained from Prof. Philipp Kahle (Tübingen, Germany) as well as background-matched control C57Bl/6 mice were bred and maintained in a temperature controlled SPF room under 12-hour light/dark cycle with free access to food and water at the Animal Facility of the Medical University of Innsbruck. All mice were genotyped by ear punch using PCR for human α-synuclein as previously described [22;23]. The oligodendroglial α-synuclein overexpression has been determined by 2', 3'-cyclic nucleotide 3'-phosphodiesterase (CNPase)/αSYN double immunofluorescence as previously shown [10;22;23]. All experiments were performed according to the EU and the Austrian legislation and with permission of the Ethics Board at the Federal Ministry of Science and Research, Austria (Permit No. BMWF-66.011/0034-II/10b/2010; BMWF-66.011/0128-II/3b/2011). All efforts were made to minimize the number of animals used and their suffering. Male mice in the third postnatal month (PM3) were used for the experiments.

### Motor behavior

Behavioral tests were performed blindly to the genotype according to validated procedures.

Spontaneous open field locomotor activity: The horizontal and vertical (rearing) open field activity was recorded for a period of 15 minutes applying the FlexField activity system (San Diego Instruments, CA). The test session was performed in the evening (6 p.m to 8 p.m.) in dark noise-isolated room [24]. The number of counts over a 15 min test period in the horizontal and vertical plane was taken for the statistical analysis.

Beam walking test: Motor coordination, balance and bradykinesia were assessed with the method adapted from Fernagut et al. [25] by measuring the ability of the mice to traverse a narrow beam. The beams consist of two different strips of wood (each measuring 50 cm long, one was 1.6 cm and the other 0.9 cm square cross-section) placed horizontally 20 and 50 cm above the floor, respectively. During training, three daily sessions of three trials (nine crossings) were performed using the 1.6 cm square large beam. Mice were then tested using the 0.9 cm square beam. Mice were allowed to perform three consecutive trials. The time for traversing the beam as well as the number of sideslip errors was recorded on each trial and the mean traverse duration and mean number of sideslip errors during a three-trial session was kept as the variable.

Pole test: The pole test was performed according to established protocols [26]. Each mouse was habituated to the test the day before. A wooden vertical pole with rough surface, 1 cm wide and 50 cm high was applied. The mouse was placed with the head up at the top of the pole and the time for turning downwards (T_turn_) as well as the total time for climbing down the pole until the mouse reaches the floor with the four paws (T_total_) was taken in 5 trials. The best performance of all the five trials was kept for the statistical analysis.

*Grip strength*: Grip strength was defined in gram by testing the ability of each mouse to keep holding to a grid while slowly increasing the load.

DigiGait test: The stride analysis was performed applying the DigiGait^TM^ Analysis System (Mouse Specifics, Quincy, MA) as previously described [27]. Mice were placed on a transparent motor-driven treadmill belt and the gait was recorded with a high-speed digital video camera placed below the belt at speed of 25 cm/s. The collected images were analyzed with the specific DigiGait Software 9.0 (Mouse Specifics, Quincy, MA) and relevant gait parameters including stride length, stride variability and step angle were assessed.

### Neuropathological analysis

Mice were transcardially perfused with ice-cold 4% paraformaldehyde in PBS under deep thiopental anesthesia. Brains were postfixed in the same fixative overnight at 4°C, cryoprotected in 30% sucrose and slowly frozen in 2-methylbutan and kept at -80°C until further processing. Serial 40 μm sections were cut on a cryotome (Leica, Nussloch, Germany). Immunohistochemistry was performed according to previously reported protocols [28]. The following primary antibodies were applied: monoclonal anti-tyrosine hydroxylase (TH, Sigma, St. Louis, U.S.A.), monoclonal anti-DARPP-32 (BD Transduction Laboratories), monoclonal anti-glial fibrillary acidic protein (GFAP, Millipore, Tamecula, CA), monoclonal anti-Iba1 (Abcam, UK) and monoclonal anti-α-synuclein (human) (15G7, Enzo Life Sciences, Lörrach, Germany). Stereological analysis was performed applying a computer-assisted image analysis system (Nikon E-800 microscope, digital camera DXM 1200; Stereo Investigator Software, MicroBrightField Europe e.K., Magdeburg, Germany). The optical fractionator stereological method was used to estimate cell numbers including the number of TH-positive dopaminergic neurons in SN, the DARPP-32-positive neurons in striatum, as well as the number of type A, B, C and D of Iba1-positive microglia in both SN and striatum [29]. GFAP relative optical density (ROD) in SN and striatum was determined as previously described [30]. To assess cell death TUNEL staining was performed with the In Situ Cell Detection Kit, POD (Roche) according to the manufacture’s protocol. Brain sections from 12 months old MSA mice were used as a positive control. Samples were coverslipped with IS mounting medium (Dianova, Hamburg, Germany) and TUNEL positive nuclei were detected in the range of 515–565 nm (green) with a Leica fluorescence microscope DMI 4000B provided with Digital Fire Wire Color Camera DFC300 FX and Application Suite V3.1 by Leica.

Statistical analysis of behavioral and neuropathological data to compare control and transgenic MSA mice was done by t-test analysis with GraphPad Prism 5.03 software. Statistical significance was set at p<0.05. Data were presented as mean ± S.E.M.

### RNA preparation and processing

Mice were sacrificed by cervical dislocation, brains were quickly extracted and SN and striatum were dissected on ice. Three pools of SN and striatum, respectively, from 5 mice each were prepared and frozen in liquid nitrogen. Total RNA was isolated using Tri-Reagent (Sigma-Aldrich, St. Louis, Missouri, USA) according to the manufacturer's instructions and dissolved in DEPC-water. Quantity and quality of RNA preparations was determined by 2100 Bioanalyzer (Agilent Technologies, Palo Alto, CA) measurement.

RNA-seq analysis of mRNA expression: For RNA-seq analysis, RNA was further purified using the RNA Clean&Concentrator Kit-25 (Zymo Research, Irvine, CA). RNA was then submitted to polyA-enrichment (Dynabeads® mRNA Purification Kit, Life technologies) using 6 μg of total RNA from each pooled sample. With the polyA-enriched RNA as input, libraries were generated with the Ion Total RNA-Seq Kit v2 (Ion torrent, Life Technologies) and barcoded following the manufacturer’s instructions, except that the final bead-based size selection was adjusted to favor longer fragments (average read lengths >150 bp). The libraries were sequenced on the Ion Proton Sequencer (Ion Torrent, Life Technologies), generating on average 90 million reads per sample.

Microarray analysis of mRNA expression: 300 ng total RNA from SN of MSA transgenic mice and control mice was processed using the Gene Chip WT reagent kit (Affymetrix) according to the manufacturer’s instructions. In brief, RNA was amplified, reverse transcribed into biotinylated single stranded cDNA and finally hybridized to whole-transcript Mouse Gene ST 2.0 microarrays (Affymetrix). Gene chips were washed and stained using fluidic station 450 (Affymetrix), fluorescence signals were recorded by an Affymetrix scanner 3000 and image analysis was performed with the GCOS software (Affymetrix). Robust-multiarray average (RMA) [31] was applied for normalization. Intensities were log_2_-transformed and filtered for probe set with interquartile range >0.35. Data preprocessing was done using R and the Bioconductor package oligo [32].

Microarray analysis of miRNA expression: Labeling was performed by the Mercury LNA microRNA Hi-Power Labeling Kit from Exiqon. The standard protocol from Exiqon was employed with minor modifications. Briefly, 1 μg of total RNA per sample was dephosphorylated, split into two aliquots, which were subsequently fluorescently labeled with either Cy3 or Cy5 dyes to be used in dye swap experiments. Labeled samples were hybridized to miRCURY LNA miRNA Array 7^th^ generation microarray (Exiqon Inc., Woburn Massachusetts, USA) using the Tecan HS400 Pro device (Tecan Group Ltd., Männedorf, Switzerland). Experiments were performed in dye swap pairs with three biological replicates from transgenic MSA mice and age- and sex-matched control mice, for both SN and striatum. The hybridization and washing of microarray slides was performed according to the manufacturer’s protocol. Upon washing, slides were dried by nitrogen gas at 23°C for 5 min. Scanning was performed immediately following hybridization at a resolution of 5 μm using the Tecan Powerscanner device (Tecan Group Ltd., Männedorf, Switzerland). Microarray scans were quantified using the data analysis software ArrayPro 6.3.

### Differential expression analysis of RNA-seq and mRNA microarray data

RNA-seq raw reads were trimmed for residual adapter sequences and low quality sequences were removed using cutadapt [33] and FASTX-Toolkit (http://hannonlab.cshl.edu/fastx_toolkit/index.html). Reads were then mapped to the *Mus musculus* genome (ENSEMBL release 38.78) using the STAR mapper for RNA sequences [34] and bowtie2 [35] in local mode for the unmapped reads. Mapped sequencing reads were analyzed by the HTSeq framework with the predefined parameter set [36]. Differential expression analysis of control versus transgenic MSA mice of both, striatum and SN samples, was performed by employing the DESeq2 package with predefined parameters [37]. Genes with an adjusted p-value below 0.1 after multiple testing corrections were considered statistically significant [38].

For microarray data differential gene expression was tested by a moderated t-test using the *limma* package [39]. For both methods genes with an adjusted p-value < 0.1 after multiple testing corrections were considered statistically significant [38]. In order to obtain a single list of regulated mRNA candidates in SN, differentially expressed mRNAs from microarray and RNA-seq experiments were combined for the following miRNA-mRNA correlation analysis.

### Differential expression analysis of miRNA microarray data

Gene expression analysis of both SN and striatum microarray datasets was performed by employing the R Bioconductor platform [40], in particular the *limma* package [39] and the MmPalateMiRNA package [41], which provide several functions for miRNA microarray analysis. For annotation, the default annotation file for the “miRCURY LNA miRNA Array 7^th^ generation hsa, mmu & rno” was employed as provided by Exiqon (Inc., Woburn Massachusetts, USA). Normalization was carried out by (a) calculating a local linear regression based on the print tip groups within arrays and (b) employing quantile normalization between arrays [42]. Normalization was based on the net intensity values ((raw intensity)–(local background)) as calculated by the ArrayPro 6.3 software (Tecan Group Ltd., Männedorf, Switzerland). Subsequently, normalization was inspected by quality metrics [43]. A non-specific filtering step was conducted by employing the “rowSDS” and “shorth” functions of the genefilter package (version 1.48) for filtering by variance. In addition, all candidates that were missing an annotation in mouse were removed. Following, differential expression analysis was performed by calculating a linear model for each miRNA according to the guidelines for simple dye swap experiments [39]. Duplicated spots were considered in the linear model fit. This model was then employed to obtain test statistics by the empirical Bayes method providing stable estimations for the sample variance of a small number of arrays [44]. All differentially expressed miRNAs with an adjusted p-value < 0.1 after multiple testing corrections as proposed by Benjamini and Hochberg were considered statistically significant [38].

### Quantitative RT-PCR analysis

For mRNA analysis total RNA was reverse transcribed and used for real time PCR as described before [45]. PCR primer sequences are stated in S1 Table. Data were normalized against *Gapdh* and differences between control and MSA samples were calculated from ΔΔC_T_ values. The assay was performed in three biological replicates. For miRNA analysis total RNA was retro-transcribed using the miScript II RT Kit (Qiagen) following the manufacturer´s instructions. 2 μg of total RNA was used as input per RT reaction. RT-PCR was performed in a ViiA7 real-time PCR sytem (Applied Biosystems) with the miScript SYBR Green PCR Kit (Qiagen) using specific forward primers corresponding to the mature miRNAs and the miScript universal primer provided with the kit (S1 Table). Ct values were normalized to that of U6 snRNA and differential expression between MSA and control samples was calculated using the ΔΔCt method. The assays were performed in technical triplicates.

Two-way ANOVA with-Bonferroni correction for multiple testing was applied to identify significant differences in the expression of miRNAs or mRNAs between control and MSA mice. Results were considered significant at p<0.05.

### Enrichment analyses

Gene ontology (GO) enrichment analysis of differentially expressed mRNAs was performed separately for down- and up-regulated genes. Data from the RNA-seq analysis in striatum were utilized as input gene list and all annotated genes resulting from the RNA-seq analysis were employed as background gene list. For SN, data from both RNA-seq and microarray analysis were combined to generate the differentially expressed gene list and also the background gene list. GO enrichment analysis was performed by employing the R package GOStats (version 2.32.0) with predefined parameter set [46] on the available ontologies, i.e. biological process (BP), molecular function (MF) and cellular compartment (CC). The resulting significantly represented enriched GO-Terms (p<0.05) were filtered by allowing only the inclusion of GO-terms that contain at least two differentially expressed genes. Next, GO-terms were further processed by utilizing REVIGO [47], a program that clusters GO-terms by their relationship and p-values. The reference database for mouse was chosen for calculating GO-term sizes and 0.5 was chosen as threshold for the similarity of GO-terms as parameter set for REVIGO. KEGG pathway enrichment analysis was performed on differentially expressed mRNAs in striatum and SN by employing the R package clusterProfiler (version 2.3.3) with q-value cutoff of 0.1 [48].

MiRNA family information was obtained from mirBase [49]. Enrichment analysis of differentially expressed miRNAs from both striatum and SN was performed separately by utilizing the R package gage (2.16.0) with predefined parameters [50].

### miRNA-mRNA correlation analysis

Genes which were predicted to exhibit 3’ UTR target sites for differentially expressed miRNAs from striatum and SN were downloaded from the mirWalk 2.0 website [51] by choosing a p-value cutoff of 0.1. In addition, predicted target genes of other prediction programs i.e. RNA22 [52], miRanda [53] and TargetScan [54] were obtained from the mirWalk 2.0 website. Validated miRNA target genes for all differentially expressed miRNAs were downloaded from mirWalk 2.0 and from miRTarBase[55]. The calculation of the correlation coefficient between mRNAs and miRNAs was conducted by employing the Pearson correlation analysis in each region (SN and striatum). Thereby, the expression values of differentially expressed miRNAs and mRNAs were utilized. Since there were only two MSA samples (pools of 5 brains each) present in RNA-seq analysis, the existing third control sample was ignored. For each miRNA, the correlation of all differentially expressed mRNAs was calculated and complemented with predicted and validated target information. In SN, correlation values were calculated separately for data obtained by RNA-seq and mRNA microarray analyses, respectively. These analyses resulted in a list of miRNAs containing information about their predicted target genes and correlation values. This list was filtered for miRNA-target interactions, which (i) were predicted by mirWalk 2.0 with a p-value < 0.1, or (ii) were predicted by at least two prediction programs, or (iii) feature a validated target gene. In all cases, a minimum correlation value of -0.3 was required. The filtered list can be found in S10 Table.

Array design and raw expression files have been deposited in ArrayExpress (E-MTAB-3985, E-MTAB-3986, E-MTAB-3993).

## Results

### Neuropathological and functional characterization of the MSA transgenic mouse model in the pre-motor stage of disease

**Fig 1:**
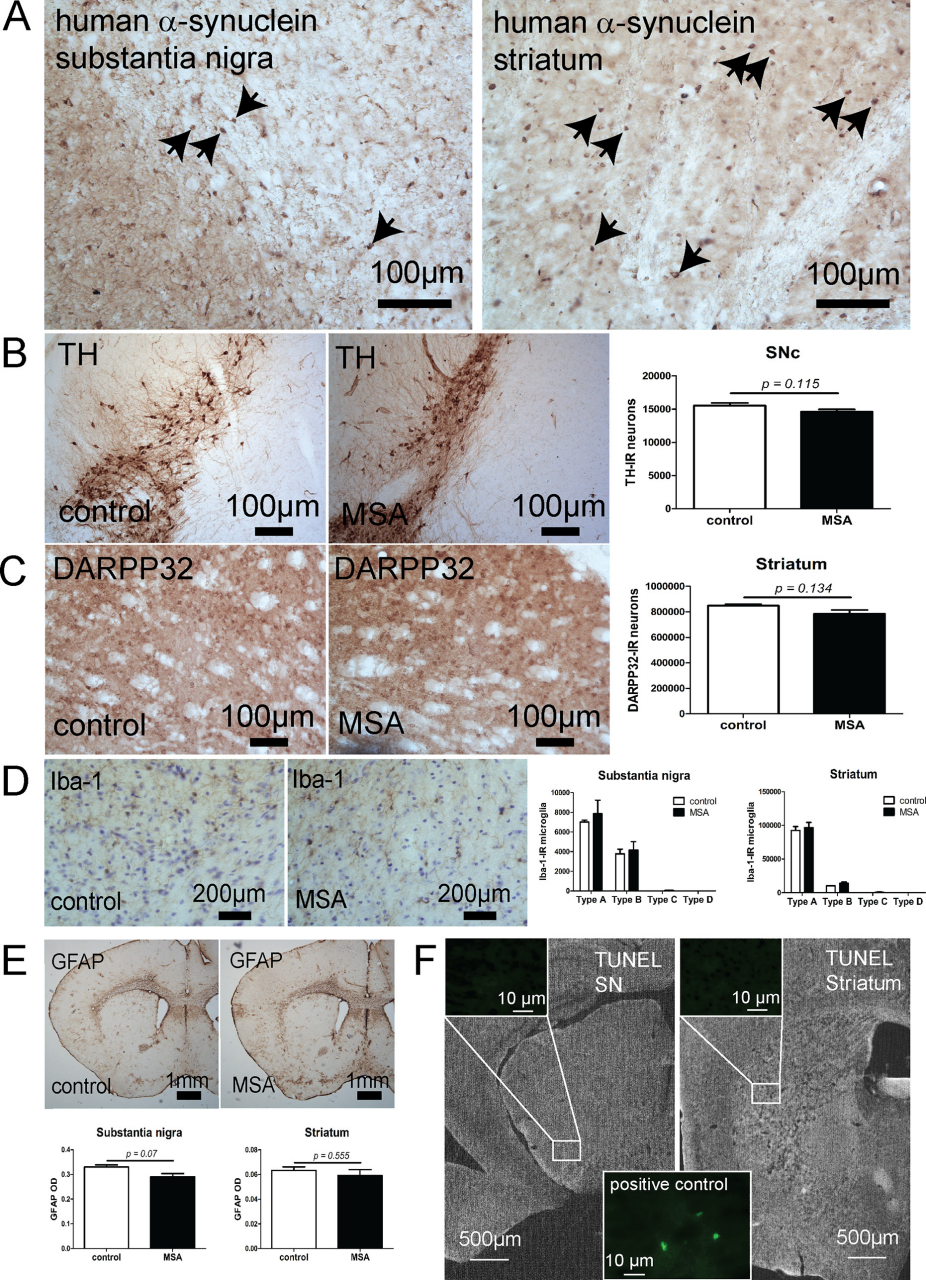
Neuropathological and behavioral characterization of a mouse model of a pre-motor stage of MSA. (A) Human α-synuclein overexpression in MSA transgenic mice resulted in α-synuclein accumulation in oligodendrocytes (arrows) detectable both in substantia nigra and striatum. (B) No dopaminergic neuronal loss was identified in the pre-motor stage in substantia nigra of MSA mice (n = 6) as compared to controls (n = 4) by stereological determination of the number of tyrosine hydroxylase (TH)-immunoreactrive (IR) neurons. (C) No GABAergic medium spiny neurons loss was identified in the pre-motor stage in striatum of MSA mice (n = 6) as compared to controls (n = 4) by stereological determination of the number of DARPP-32-IR neurons. (D) Iba-1-IR was used to determine the number and activation status of microglia (type A, B, C, and D [29]) in MSA (n = 3) and control mice (n = 3). No significant differences were detected between the groups with predominant representation of type A resting microglia in both substantia nigra and striatum. (E) GFAP-immunohistochemistry was used to determine the level of astroglial activation in MSA (n = 5) and control mice (n = 3) in substantia nigra and striatum. No significant differences were identified between the groups. Statistical analysis of the neuropathological data to compare control and transgenic MSA mice was done by t-test analysis with GraphPad Prism 5.03 software. Statistical significance was set at p<0.05. Data are presented as mean ± SEM. (F) TUNEL staining detected no cell death in SN and striatum of PM3 MSA mice. As a positive control we applied aged PM12 MSA mice (an age when detectable neuronal loss is recorded) that demonstrated positive TUNEL staining.

In the MSA transgenic mouse model used in this study, neurodegeneration in SN and striatum accompanied by GCI-like pathology, similar to the human disease, has been detected at an older age (over 4 months) [13]. When we analysed PM3 transgenic α-synuclein expression, we found accumulation of human α-synuclein in oligodendrocytes mimicking the GCI pathology of human MSA (Fig 1A). Furthermore, RNA expression analysis revealed a 260-fold increase of *SNCA* expression in SN and a 163-fold increase in striatum (see S2 and S3 Tables). Despite the presence of GCIs, no neuronal loss was identified in SN or striatum, respectively, at this stage (Fig 1B and 1C). Gliosis has been described in the degenerating brain areas in both symptomatic human MSA [56;57] and after four months of age in the MSA mouse model [11]. However, no changes in the morphological microglia activation profile between control and MSA mice in PM3 were identified in the striatum and SN (Fig 1D) as well as no astroglial activation was detected (Fig 1E). To identify whether cell damage resulting with cell death was detectable at this stage TUNEL staining was performed. No indication of cell death in MSA mice in the pre-motor stage of disease was detected neither in the SN, nor in the striatum (Fig 1F). The absence of neuronal loss was reflected by the preserved motor performance of MSA transgenic mice as compared to control animals at this age (Table 1). In summary, the motor and neuropathological analysis of the MSA transgenic mouse showed that, at the young age (PM3), the model replicates an early pre-motor phase of MSA-like pathology with presence of α-synuclein accumulation in oligodendrocytes but lack of neuronal loss in SN and striatum (two brain regions that are later affected by neurodegeneration) and respectively absence of motor deficits.

**Table 1:** Motor analysis of MSA versus age-matched control mice.

motor parameter	type	n	mean±SEM	t	p
Open field rearing (counts)	control	13	166.6±20.23	0.9581	0.3462
	MSA	17	195.2±21.02		
Open field horizontal activity	control	13	2951±162.6	1.552	0.132
(counts)	MSA	17	3306±156.9		
T-turn in pole test (seconds)	control	14	0.933±0.088	0.3411	0.7355
	MSA	17	0.898±0.059		
T-total in pole test (seconds)	control	14	6.503±0.357	0.6834	0.4998
	MSA	17	6.115±0.422		
Beam walking time (seconds)	control	14	3.29±0.159	1.145	0.2614
	MSA	17	3.04±0.155		
Beam walking errors	control	14	0.143±0.063	0.144	0.8867
	MSA	17	0.157±0.072		
Grip strength (g)	control	14	150.7±4.318	0.1397	0.8899
	MSA	17	151.8±5.815		
Stride length (cm)	control	8	5.819±0.126	0.8371	0.4157
	MSA	9	5.972±0.131		
Stride length variablity (CV%)	control	8	14.08±1.492	0.7593	0.4594
	MSA	9	16.51±2.7		
Step angle (degree)	control	8	63.00±2.022	1.977	0.0667
	MSA	9	56.78±2.357		

Motor behavior was analyzed by several behavioral tests. Comparison between MSA and control mice by t-test detected no functional differences.

To study whether changes at the molecular level in the striatonigral continuum precede the onset of neurodegeneration and motor symptoms, we investigated MSA mice at a pre-motor stage of the disease and wild-type healthy controls by gene expression profiling. To this end, we examined global mRNA as well as miRNA expression patterns in striatum and SN using a combination of RNA-seq and microarray-based miRNA and mRNA profiling and performed correlation analyses to decipher potential miRNA-mRNA regulatory networks (S1A Fig).

### Significant alterations in mRNA profiles in striatum and SN of early stage MSA mice

**Fig 2:**
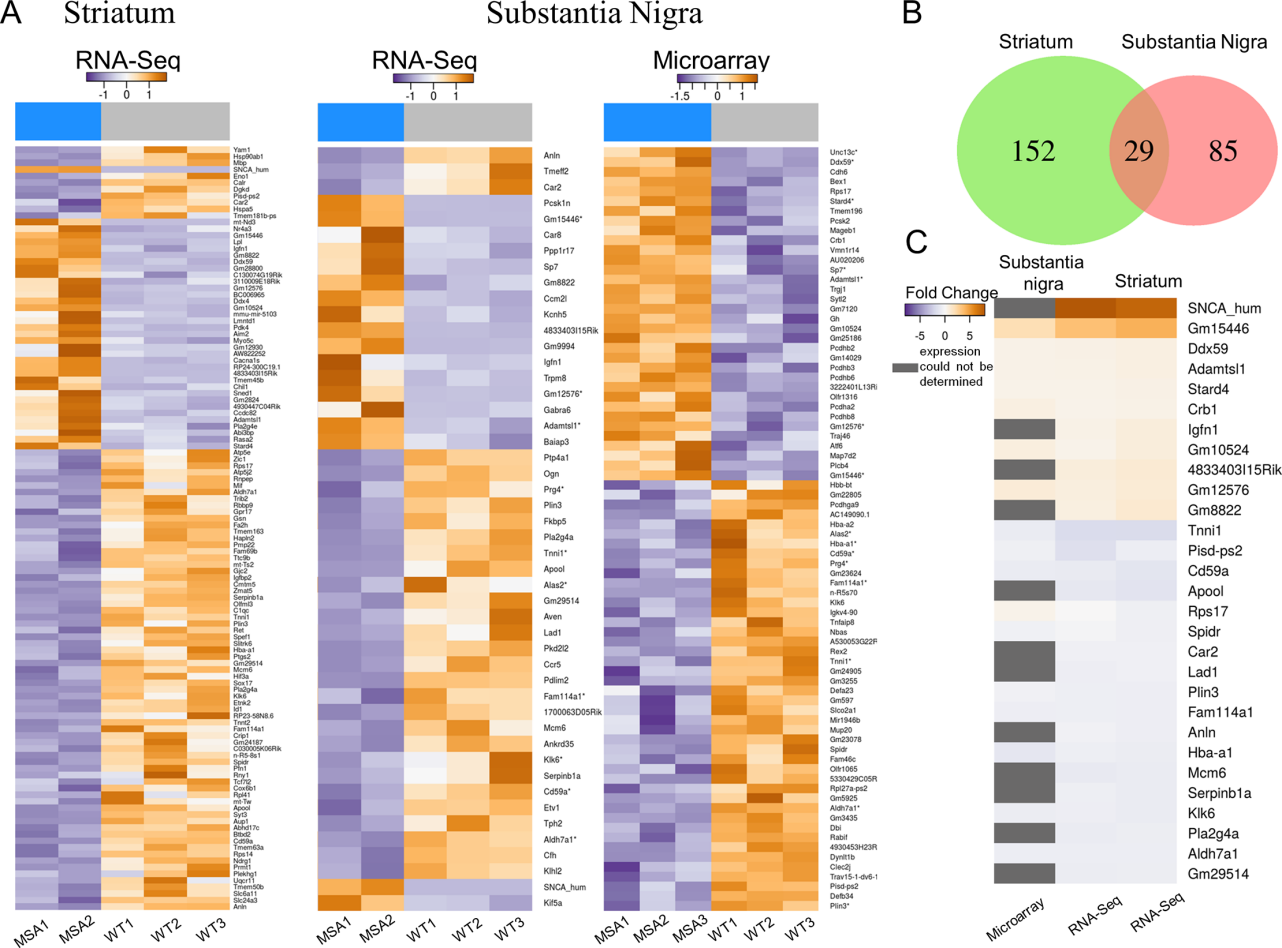
Differential expression of mRNAs in a mouse model of pre-motor stage MSA. (A) Heatmaps represent significantly differentially expressed genes of RNA-seq (striatum, SN) and microarray (SN) analyses. For each gene (row), the log2-transformed change of the expression value in each sample to the average expression value over all samples is shown. Columns represent individual replicates grouped into MSA and control (WT) samples indicated by the blue (MSA) and grey (WT) bars at the top of the heatmaps. The color gradient indicates the expression change from negative to positive. The asterisks following gene names indicate overlapping genes between microarray and RNA-seq analyses in SN. (B) Venn diagram illustrating the number of overlapping differentially expressed mRNAs between SN and striatum tissue in MSA mice. (C) Heatmap highlights log_2_-transformed fold changes of mRNAs overlapping between striatum und SN. Down-regulated mRNAs are indicated by a blue color gradient, whereas up-regulated miRNAs are indicated by an orange color gradient. mRNA with expression signals below background in the microarray experiment are highlighted in gray. From left to right, microarray and RNA-seq analysis results of SN and RNA-seq analysis of striatum are shown. Differential expression analysis of control versus transgenic MSA mice of both, striatum and SN samples, was performed by employing the DESeq2 package with predefined parameters [37]. Genes with an adjusted p-value below 0.1 after multiple testing corrections were considered statistically significant [38]. For microarray data differential gene expression was tested by a moderated t-test using the *limma* package [39]. For both methods genes with an adjusted p-value < 0.1 after multiple testing corrections were considered statistically significant [38].

To examine the expression pattern of mRNAs from MSA transgenic mice in a pre-motor phase of the disease, we performed RNA-seq for striatum samples as well as RNA-seq and microarray analysis for SN of MSA mice and healthy age-, sex- and background-matched controls. These experiments revealed 181 differentially expressed mRNAs in striatum, 119 of which were down-regulated while the expression of 62 mRNAs was up-regulated in MSA mice (Fig 2A and S2 Table). In SN, of the 48 differentially expressed mRNAs identified by RNA-seq analysis, 30 mRNAs were down-regulated while expression of 18 genes was up-regulated (Fig 2A). By microarray analysis, we detected 44 down-regulated and 34 up-regulated mRNAs (Fig 2A). Significant differential expression of 12 mRNAs was found by both methods, while the remaining de-regulated mRNAs showed significant changes detected by either one of both methods (S1B Fig). To verify the accuracy of the techniques we performed: i) correlation analysis of mRNA expression acquired by RNA-seq and microarray by Bland-Altman plot ([58], S1C Fig), and ii) RT-PCR analysis of representative mRNAs (S4 Fig). Upon closer investigation, we noted that the non-overlapping mRNAs, which were found to be differentially expressed by employing either RNA-Seq or microarray, exhibited similar expression fold-changes in the respective alternative method but were not detected as differentially expressed in the analysis because they missed the threshold for statistical significance (p<0.1) (S1D Fig). Since even small changes in mRNA expression might be significant at the early stage of the disease, but might differently be identified by two fundamentally different analysis methods, such as RNA-seq and microarray, we decided to combine the results from both analyses giving at total of 114 de-regulated genes in SN (S3 Table), thereby accepting potentially lower stringency standards.

When comparing mRNA expression profiles of striatum and SN in pre-motor MSA mice, we identified 29 mRNAs, which showed differential expression in both tissues (Fig 2B). Thereby, only one gene, i. e. ribosomal protein S17 (*Rps17*), showed an opposite expression pattern in striatum and SN tissue (Fig 2C). Furthermore, 152 genes were differentially expressed in the striatum but not in SN, while another 85 genes were differentially expressed in SN, but not in the striatum of pre-motor MSA mice. These results point towards region-specific differences in transcriptome de-regulation in SN and striatum, which may be linked to differences in their vulnerability.

### Functional enrichment analysis of differentially expressed genes in the mouse model of pre-motor MSA

In order to get deeper functional insights into the role of the differentially expressed mRNAs, we examined the potential enrichment of GO terms for biological processes for up- and down-regulated genes, respectively, using R package GOStats (version 2.32.0).

For the striatum of MSA mice we identified 191 enriched functional categories (p<0.05; S4 Table). Several GO terms were highly relevant to suggested human MSA disease pathways [59–63]. To provide further objective and unbiased analysis of the data, REVIGO clustering based on gene relationships and p values in the striatum was performed (http://revigo.irb.hr/). This analysis revealed clusters of dysregulated genes related to 6 major modules and categories (S5 Table) including:

1. oligodendroglial dysfunction;
2. protein handling;
3. dysfunctional metabolism (including monocarboxylic acid biosynthetic process, cellular lipid metabolic process and lipid biosynthetic process);
4. disrupted transmembrane transport;
5. altered inflammatory and immune responses.
6. cell death.

In addition, KEGG pathway enrichment analysis for striatum revealed differentially expressed genes which are involved in related neurological disorders such as AD, PD or Prion disease and genes linked to disturbed protein processing in endoplasmic reticulum, lipid metabolism, immune response/antigen presentation, ribosomal function and oxidative phosphorylation (S1E Fig).

Functional enrichment analysis for de-regulated genes in SN of MSA mice in a pre-motor stage of the disease identified 28 significantly enriched categories (p<0.05) (S6 Table). REVIGO analysis (http://revigo.irb.hr/) of the GO-terms linked to differentially expressed genes in the SN of MSA mice identified two major clusters (S7 Table) linked to the modules:

1. altered immune and inflammatory responses;
2. disrupted ion (including oxygen) homeostasis and oxidative stress.

KEGG pathway analysis in SN of pre-motor MSA mice (S1F Fig) indicated differentially regulated enriched pathways linked to parasitic diseases like African trypanosomiasis and Malaria with strong involvement of the immune system, to nitrogen metabolism, which may be associated with neurotoxicity [64], and to long-term depression possibly related to changes in synaptic transmission [65].

### Differential expression profile of miRNAs in striatum and SN of pre-motor MSA transgenic mice

**Fig 3:**
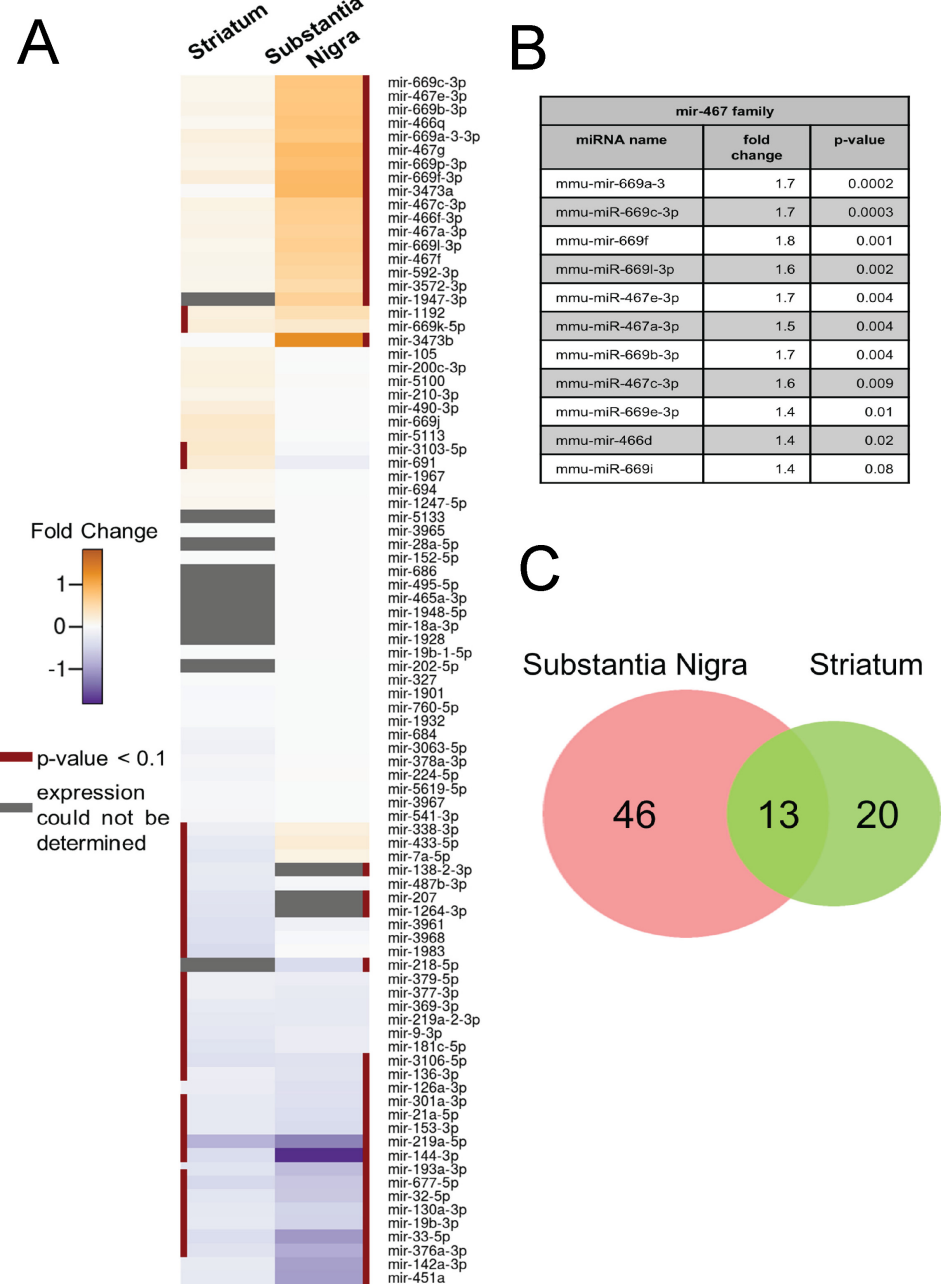
Differential expression of miRNAs in a mouse model of pre-motor stage MSA. (A) Heatmap shows expression changes of miRNAs of striatum (left) and SN (right). miRNAs with statistically significant (adjusted p<0.1) changes are indicated by a red line on the side. Gray boxes designate miRNAs with expression signals below background. The color gradient shows positive and negative log_2_-transformed fold changes in orange and blue color, respectively. (B) Fold change and adjusted p-value of the miRNAs of the mir-467 family. (C) Venn diagram illustrates the overlap of differentially expressed miRNAs between SN and striatum in MSA mice. Differential expression analysis was performed by calculating a linear model for each miRNA according to the guidelines for simple dye swap experiments [39]. Duplicated spots were considered in the linear model fit. This model was then employed to obtain test statistics by the empirical Bayes method providing stable estimations for the sample variance of a small number of arrays [44]. All differentially expressed miRNAs with an adjusted p-value < 0.1 after multiple testing corrections as proposed by Benjamini and Hochberg were considered statistically significant [38].

Since miRNAs are known to be potent regulators of gene expression, we sought to determine, whether potential changes in miRNA levels might be correlated to the observed deregulation of mRNA expression in striatum and SN of young MSA mice. To this end, we employed the Exiqon miRCURY LNA miRNA array to investigate global miRNA expression patterns in the same MSA- and control mouse samples that were used for mRNA profiling. These experiments revealed 33 miRNAs whose expression was de-regulated in striatum (S8 Table). Among the differentially expressed miRNAs, four showed up-regulation in their expression, while the expression of 29 miRNAs was down-regulated (Fig 3A and S8 Table).

The analysis of miRNA expression patterns in SN revealed a higher number of deregulated miRNAs in SN than in striatum. Thereby, a total of 59 differentially expressed miRNAs was observed with 29 being up-regulated and 30 down-regulated (Fig 3A and S9 Table). Analysis of differentially expressed miRNAs showed significant enrichment (p<0.001) of the miRNA family mir-467 among the up-regulated miRNAs (Fig 3B). Furthermore, comparison of striatum and SN tissues revealed 13 miRNAs, whose expression was down-regulated in both brain areas in MSA mice compared to controls (Fig 3C).

### Predicted regulatory networks of miRNA-mRNA interactions in the pre-motor MSA mouse model

**Fig 4:**
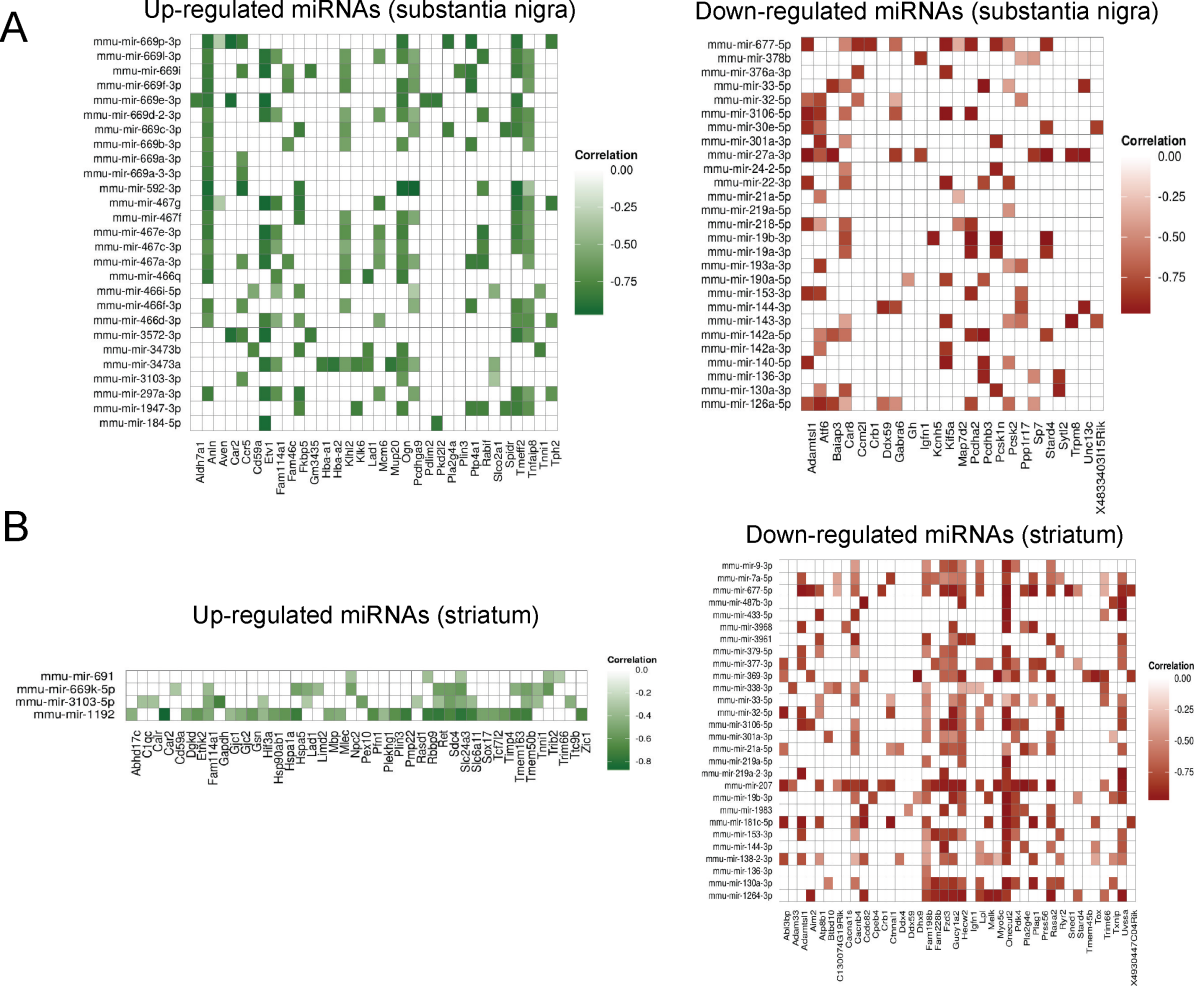
**De-regulated miRNAs and their correlation to putative target de-regulated mRNAs** in substantia nigra (A) and striatum (B) of MSA mice in a pre-motor stage of disease. Shortlisted miRNA-targets are based on 3 factors: (i) predicted in miRwalk with p-value<0.1 (ii) experimentally validated and (iii) present in at least two prediction programs.

Next, to identify potential regulatory miRNA-mRNA interactions, we performed correlation analyses of de-regulated mRNAs and miRNAs in striatum and SN. We identified 56 candidate mRNA interaction partners of 54 miRNAs in SN and 81 candidate mRNA interaction partners of 32 miRNAs in striatum of the early stage MSA mouse model. The candidate mRNA partners showed a predicted or validated 3’-UTR miRNA binding site and a negative correlation with at least one differentially expressed miRNA from our screen as well as associated to at least one significantly enriched functional category of the respective brain region (Fig 4). To verify the results, representative miRNA-mRNA predicted pairs were analyzed additionally by RT-PCR analysis (S5 Fig).

**Fig 5:**
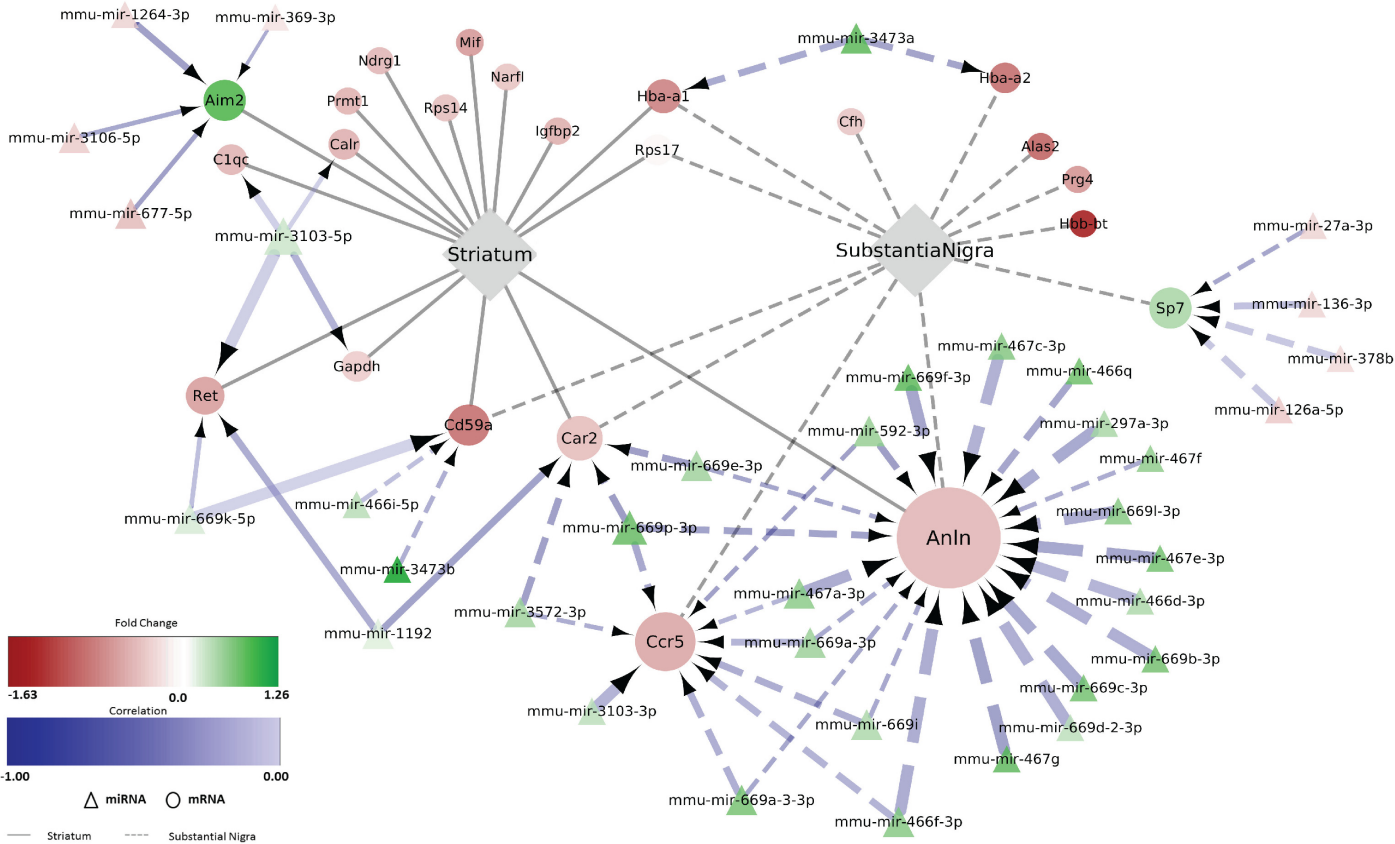
Deregulated miRNA-mRNA regulatory network to “Immune system process” in MSA mice in disease pre-motor stage. Differentially expressed miRNAs with predicted negatively correlated differentially expressed mRNA targets are visualized by employing Cytoscape (version 3.2.1). Round nodes show mRNA and triangle nodes miRNA. Node size is proportional to its degree. Fold change (log_2_ transformed) for each node is ranging from red (negative) to green (positive). Interaction arrow thickness is proportional to the number of algorithms predicting the miRNA-mRNA target 3’ UTR interaction, ranging from one to four. Differential expression of genes, in striatum and SN, such as *Anln*, *Car2*, *Cd59a*, *Hba-a1* and *Rps17*, is visualized by color corresponding to the mean fold change (exact values can be found in S2 and S3 Tables).

To assess the biological implications of the miRNA-mRNA regulatory network, we inspected these candidate interactions more closely with respect to the significantly enriched GO-categories reported above (S2 and S3 Figs). Several candidate interactions of up-regulated miRNAs and down-regulated target genes were assigned to the category “immune system process”, which was found as one of the major enriched GO terms in our mRNA profiling data both in striatum and SN (Fig 5). Among those genes are, for instance, *Anln*, *or CD59a*, which we found to be down-regulated in both SN and striatum. Thereby, Anillin (*Anln*) was predicted to be regulated by the highly enriched mir-467 cluster. *Anln* encodes an actin-binding protein involved in dynamic reorganization of the actin cytoskeleton that may link to the control of TLR4 mediated phagocytosis [66] and to the regulation of epithelial junctions [67]. *CD59a* encodes a protein that is involved in the regulation of the complement cascade and recent studies in knock-out mice have suggested its involvement in pathological tau accumulation [68]. By contrast, some predicted de-regulated miRNA-mRNA pairs of this category were only found in one of the two brain areas. The upregulation of *Aim2* in the striatum but not the SN of MSA mice was predicted to be linked to the concomitant down-regulation of several miRNAs (Fig 5). AIM2 is an interferon-inducible protein which may induce increased release of inflammatory cytokines and inflammasome-mediated cell death [69]. Alternatively, *Ccr5* was down-regulated only in the SN of MSA mice and predicted to be controlled by several concomitantly up-regulated miRNAs. *Ccr5* that encodes a chemokine receptor and its dysfunction has been proposed to associate with disruption of the phagocytic activity of macrophages [70].

**Fig 6:**
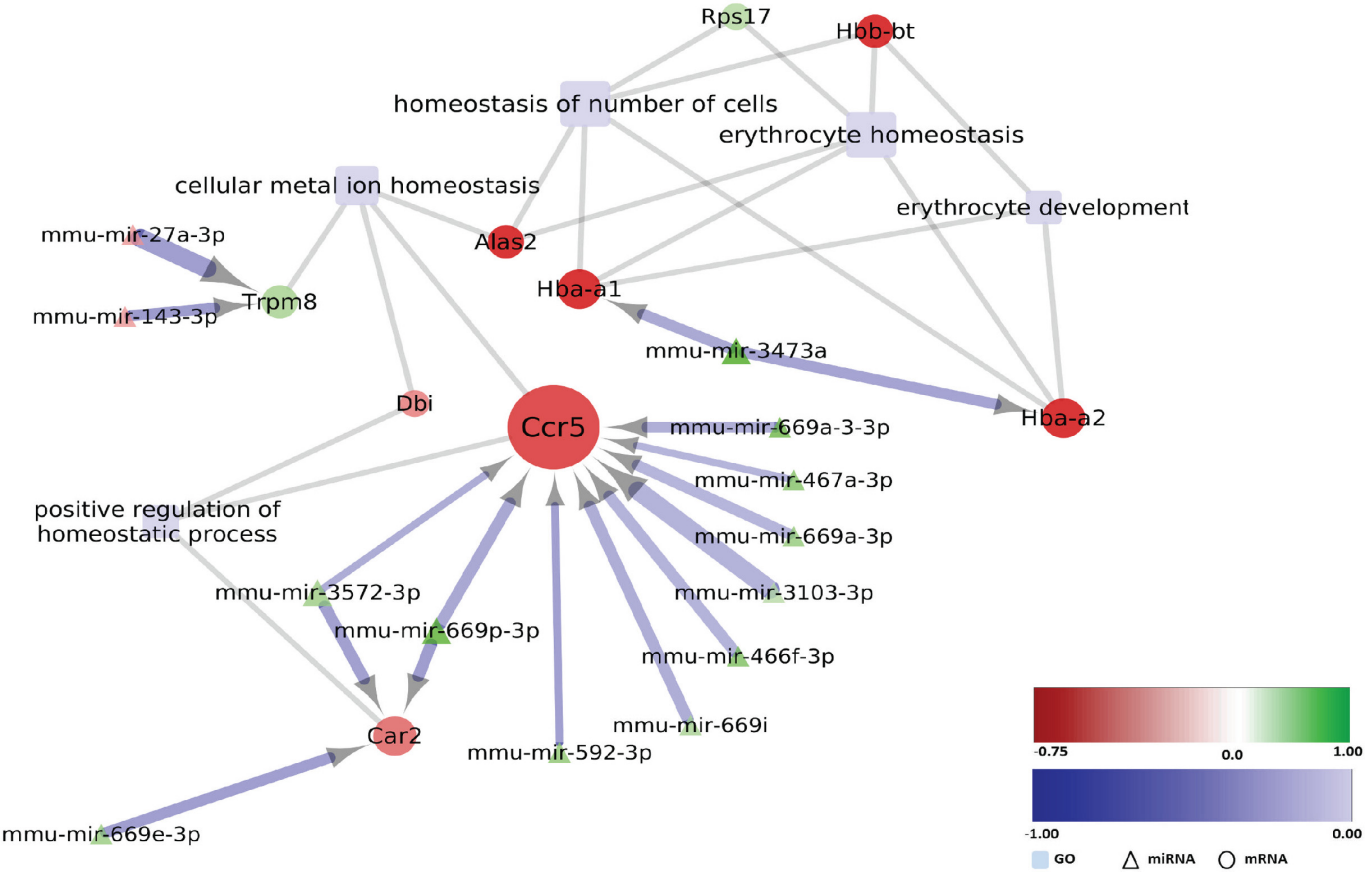
Deregulated miRNA-mRNA regulatory network to “Homeostasis/oxidative stress” in SN of MSA mice in a pre-motor stage. Differentially expressed miRNAs with predicted negatively mRNA targets assigned to the indicated GO-terms (light blue rectangles) are visualized by employing Cytoscape (version 3.2.1). Round nodes designate mRNA and triangle nodes miRNA. Node size is proportional to its degree. Fold change (log_2_ transformed) for each node is ranging from -0.75 (red) to 1 (green). The shade of blue color of the interaction arrows indicates the degree (range -1.00–0.00) of negative correlation between miRNA-mRNA target 3’ UTR interaction. Interaction arrow thickness is proportional to the number of algorithms predicting the miRNA-mRNA target 3’ UTR interaction, ranging from one to four.

In the early pre-motor disease stages of the MSA mouse model, a major cluster of genes with differential expression and related to it GO biological processes, were linked to disrupted homeostasis and oxidative stress in the SN. Some significantly down-regulated genes in this cluster (e.g. *Ccr5*, Chemokine (C-C Motif) Receptor 5; *Car2*, carbonic anhydrase 2; *Hba-a and*, *Hba-a2*, hemoglobin alpha, adult chain 1 and 2) are putative targets of up-regulated miRNAs in the region suggesting a possible miRNA-mRNA network in the SN associated with the mediation of oxidative stress in the early disease stages (Fig 6). Interestingly, some of the differentially expressed genes (e.g. *Ccr5*) were assigned to the both REVIGO cluster categories “immune and inflammatory response” and “disrupted ion homeostasis and oxidative stress” reflecting the interconnected nature of these processes. In the striatum, REVIGO clustering did not provide strong evidence for a leading role of disrupted homeostasis and oxidative stress in striatum in the early pre-motor stages of disease in the MSA mouse model (S5 Table), however differentially expressed genes associated with GO-terms like “response to oxidative stress”, “response to decreased oxygen levels” and “cellular response to nitrogen compound” (e.g. *Pdk4*, Pyruvate Dehydrogenase Kinase, Isozyme 4; *Ryr2*, Ryanodine receptor 2; *Melk*, Maternal Embryonic Leucine Zipper Kinase; *Txnip*, Thioredoxin Interacting Protein) (see S4 Table) provide putative targets of de-regulated miRNAs in the region (Fig 4B). These data may indicate that—unlike in the SN—in striatum, miRNA-mRNA interactions to regulate the oxidative stress status of cells may be less significant in the early disease stages but may gain in importance during disease progression (see also Discussion).

**Fig 7:**
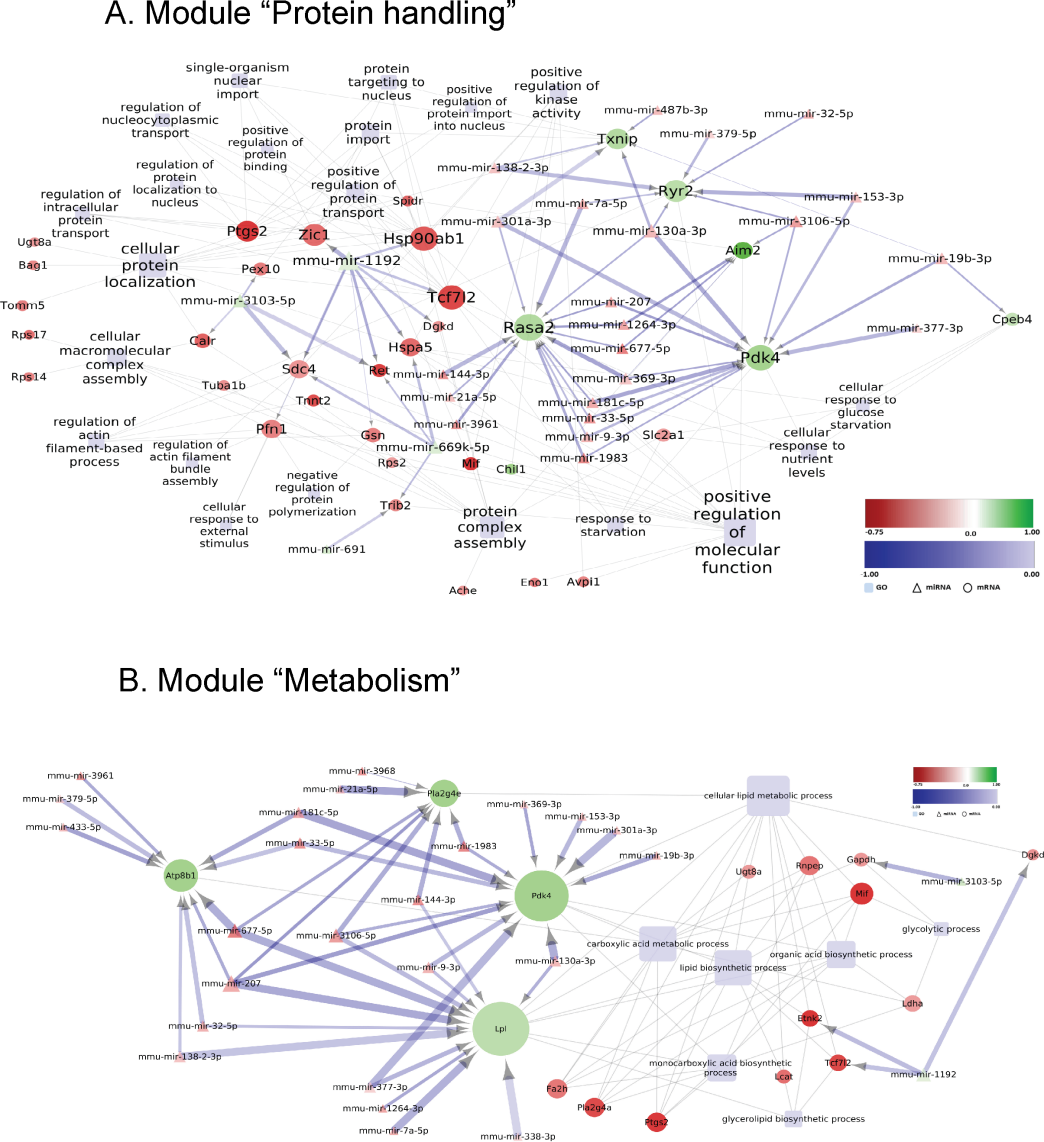
**Deregulated miRNA-mRNA regulatory network in the striatum of MSA mice in pre-motor stage of disease:** Modules “Protein handling” (A) and “Metabolism” (B). Differentially expressed miRNAs with predicted negatively correlated differentially expressed mRNA targets assigned to the indicated GO-terms (light blue rectangles) are visualized by employing Cytoscape (version 3.2.1). Round nodes designate mRNA and triangle nodes miRNA. Node size is proportional to its degree. Fold change (log_2_ transformed) for each node is ranging from -0.75 (red) to 1 (green). The shade of blue color of the interaction arrows indicates the degree (range -1.00–0.00) of negative correlation between miRNA-mRNA target 3’ UTR interaction. Interaction arrow thickness is proportional to the number of algorithms predicting the miRNA-mRNA target 3’ UTR interaction, ranging from one to four.

**Fig 8:**
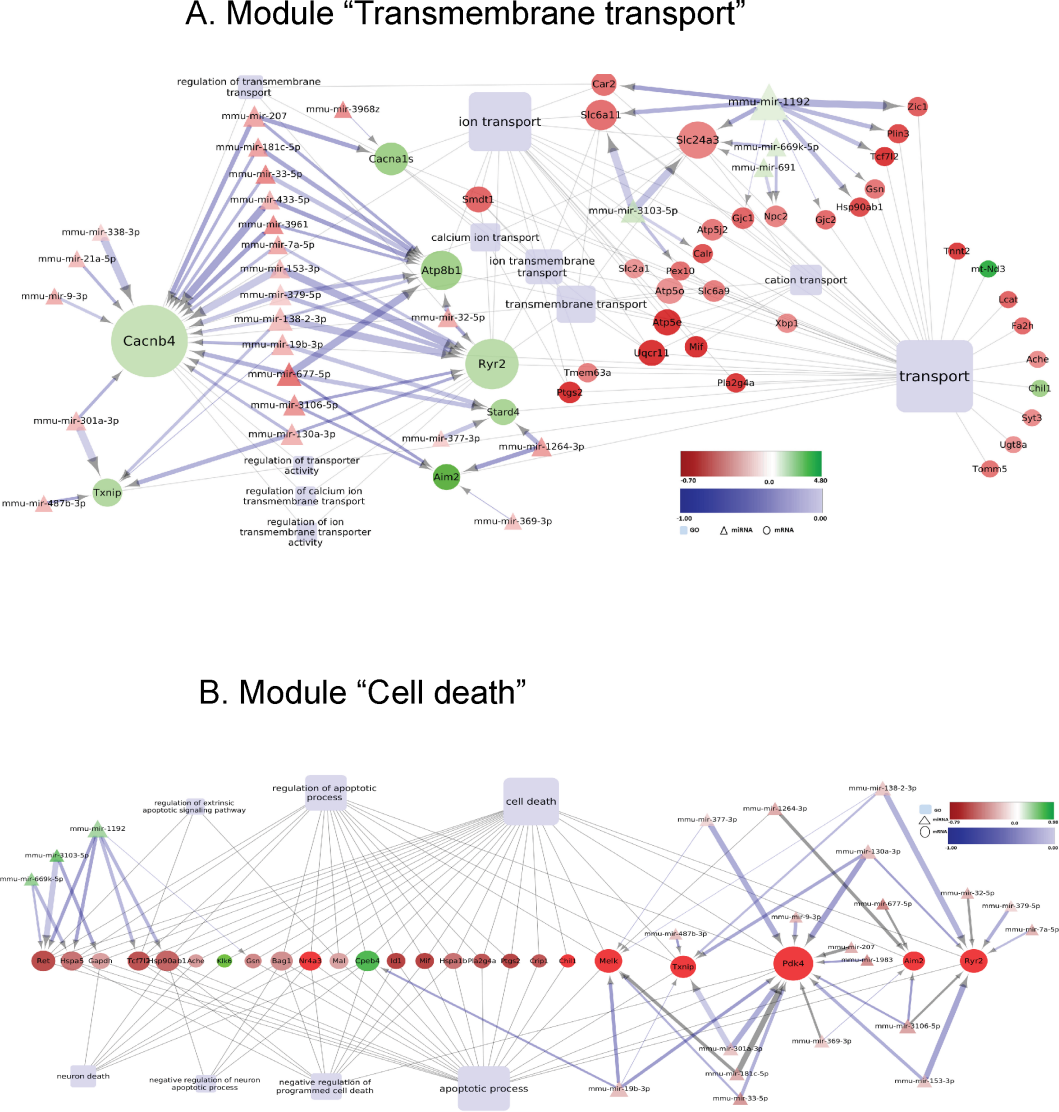
**Deregulated miRNA-mRNA regulatory network in the striatum of MSA mice in pre-motor stage of disease:** Modules “Transmembrane transport” (A) and “Cell death” (B). Differentially expressed miRNAs with predicted negatively correlated differentially expressed mRNA targets assigned to the indicated GO-terms (light blue rectangles) are visualized by employing Cytoscape (version 3.2.1). Round nodes designate mRNA and triangle nodes miRNA. Node size is proportional to its degree. Fold change (log_2_ transformed) for each node is ranging from -0.75 (red) to 1 (green). The shade of blue color of the interaction arrows indicates the degree (range -1.00–0.00) of negative correlation between miRNA-mRNA target 3’ UTR interaction. Interaction arrow thickness is proportional to the number of algorithms predicting the miRNA-mRNA target 3’ UTR interaction, ranging from one to four.

In the striatum of MSA mice in the pre-motor stage of disease, further dysfunctional biological modules were identified with variable involvement of the miRNA network in the predicted control of mRNA expression. With the current stringency of the screening, we did not find a link between miRNA modulation and oligodendroglial dysfunction/myelination (S3 Fig). Several down-regulated genes involved in the module “protein handling” (among those *Rasa2*, RAS P21 Protein Activator 2 and *Pdk4*, Pyruvate Dehydrogenase Kinase, Isozyme 4) were predicted to be controlled by several shared up-regulated miRNAs (Fig 7A). Alternatively, mmu-mir-1192 down-regulation was predicted to promote the up-regulation of genes such as *Hsp90ab1*, Heat Shock Protein 90kDa Alpha (Cytosolic), Class B Member 1; *Hspa5*, Heat Shock 70kDa Protein 5; *Tcf7l2*, Transcription factor 7-like 2; *Ret*, RET Receptor Tyrosine Kinase; *Dgkd*, Diglyceride Kinase Delta; *Sdc4*, Syndecan 4; *Zic1*, Zinc Finger Protein ZIC 1 (Fig 7A). The module “Metabolism” also harbors several genes de-regulated in the striatum of pre-motor MSA mice which represent putative targets of a considerable number of deregulated miRNAs (Fig 7B). Of note, genes with links to lipid metabolism, such as *Pdk4* (Pyruvate Dehydrogenase Kinase, Isozyme 4), *Lpl* (Lipoprotein Lipase) or *Pla2g4e* (Phospholipase A2, Group IVE) were strongly represented in this group (Fig 7B). Changes in lipid metabolism have been shown to be relevant to the human disease [62], and it is therefore intriguing to see that early alterations in this process might be caused by miRNA-mRNA network interactions. The module “Transmembrane transport” was a further category with prominent predicted interaction between dysregulated miRNAs and mRNAs. The genes *Cacnb4* (calcium channel ß4 subunit), and *Ryr2* (ryanodine receptor 2), both related to calcium transport as well as *Atp8b1* (ATPase, aminophospholipid transporter, class I, type 8B, member 1) associated with lipid transmembrane transport, were predicted to be controlled by several deregulated miRNAs (Fig 8A). Finally, some predicted miRNA-mRNA interactions grouped into the category “Cell death” in the striatum of pre-motor stage MSA mice (Fig 8B).

## Discussion

The current study represents the first evaluation of transcriptome-wide changes in mRNA and miRNA expression patterns in the SN and striatum of an MSA mouse model in a pre-motor stage of the disease and provides evidence for a dysregulation of the miRNA-mRNA regulatory network, preceding the onset of motor symptoms. This stage is characterized by very high levels of expression of the human α-synuclein transgene *SNCA* in both SN (260 fold change) and striatum (163 fold change), respectively, which corresponds to a 10-fold increased protein expression of α-synuclein (unpublished observation, NS). The accumulation of human α-synuclein in oligodendrocytes triggers a yet undefined cascade of events that leads to neuronal loss in SN and later on in striatum, which finally results in motor deterioration [12]. Therefore, the (PLP)-α-synuclein transgenic mouse replicates features of a progressive Parkinson variant of MSA with underlying SND and provides an excellent tool to address scientific questions focused on the early pre-symptomatic stages of MSA-like disease pathways [9;13].

### Transcriptome expression analysis–a window to the molecular events underlying the pathogenesis of MSA

Through transcriptome expression analysis in striatum and SN in MSA transgenic mice in a pre-motor stage of the disease we identified several major biological processes and modules with strong relevance to human MSA that appear dysfunctional in response to early α-synuclein accumulation in oligodendrocytes. Our findings indicate the role of GCI-like pathology to induce early alterations in oxygen homeostasis and oxidative stress and changes in the inflammatory/immune responses in both SN and striatum relevant to candidate pathogenic mechanisms involved in human MSA [56]. Although these mechanisms are dysfunctional in both brain structures, they appear to be predominant in the SN at this stage of the MSA disease process and appear to be associated with the earlier nigral dopaminergic neuronal loss in the MSA transgenic model, supporting the notion of higher susceptibility of SN to α-synuclein-triggered oxidative stress and inflammatory dysfunction (discussed later). Furthermore, indications of dysregulated oligodendroglial function, protein handling, transmembrane transport and metabolism, as well as switch in cell death mechanisms downstream of α-synuclein accumulation in oligodendroglia were evident in the striatum. All these processes and pathways have previously been implicated in MSA pathogenesis based on either neuropathological findings or experimental studies in later symptomatic stages of the disease [1;2;59;60;62;63;71–73].

How relevant is the transcriptome of the MSA transgenic mouse to the transcriptome in human MSA? A recent study by Mills et al. reported RNA-seq data from the grey and white matter of the frontal cortex of six MSA cases [14]. This study identified changed expression of genes related to the immune response (*HLA-A*, *HLA-B*, *HLA-C*, *IL1RL1*) and to intraneuronal oxygen homeostasis (hemoglobin complex genes *HBA1*, *HBA2* and *HBB*) consistent with findings of the current study in the transgenic MSA mouse model. The specific set of differentially expressed genes linked to altered immune response differs between both studies. This is not surprising in light of the different source of the analysed material (i.e. frontal cortex versus SN and striatum) and the stage of the disease (i.e. end-stage disease versus early onset MSA). It is encouraging, however, that both studies confirm and strengthen the role of dysfunctional immune responses in the pathogenesis of MSA even in the earlier stages of the disease. Most interestingly, both human MSA cases and the transgenic mouse model of MSA show changed gene expression of the hemoglobin protein complex. Previous studies have demonstrated the presence of hemoglobin α- and β-chain in neurons of the rodent and human brain [74–76]. It is assumed that hemoglobin may play a role in intraneuronal oxygen homeostasis, oxidative phosphorylation, iron metabolism and nitric oxide synthesis [76]. Therefore, changes in its expression may emphasize the role of oxidative stress in neurodegeneration. In support of this hypothesis and our current findings, it was shown by Ferrer et al. that neuronal hemoglobin reduction is detected in neurons with or without inclusion pathology of AD, PD and DLB brains [77].

### How similar are the pathogenic events in neuronal versus oligodendroglial α-synucleinopathy: comparison of PD and MSA pathogenesis

It is intriguing to compare the biological processes and modules involved in the pathogenesis of MSA versus PD while comparing the transcriptome analysis of striatum in MSA mice with oligodendroglial overexpression of α-synuclein to the transcriptome analysis of striatum in PD transgenic mice with neuronal overexpression of α-synuclein, both in a pre-manifestation phase of the disease [78]. While in the PD striatum strong gene dysregulation was associated with changes in signaling, synaptic function and post-synaptic signaling, we found MSA striatum to be characterized by oligodendroglial dysfunction and altered inflammatory responses. In both PD and MSA models, genes related to the regulation of lipid metabolism were affected upon *SNCA* overexpression. These data support the role of α-synuclein (of either neuronal or oligodendroglial origin) in the modulation of brain lipid turn-over as shown in human PD [79;80] and MSA [62]. Finally, regulation of apoptosis (GO:0042981) was significantly associated with the disease processes in the striatum of both PD and MSA mice. However, while in PD mice the common signature was rather in the direction of pro-survival changes consistent with the absence of neuronal cell death in the striatum in PD patients, in MSA mice the pro-apoptotic patterns (up-regulation of pro-apoptotic genes and down-regulation of survival genes) were dominant, thereby potentially laying the foundation for the later striatal neuronal loss characteristic for MSA.

### Insights into the selective vulnerability of substantia nigra and striatum

Selective vulnerability of specific brain regions in different neurodegenerative disorders remains a mystery up to date. In the current model of *SNCA* overexpression in oligodendrocytes as a trigger of selective SND, we have identified higher susceptibility of nigral dopaminergic neurons to cell death which undergo neuronal loss much earlier as striatal neurons exposed to the same noxa [13]. When comparing the differentially expressed genes in the transcriptome analysis of SN and striatum of MSA transgenic mice, we identified a proportion of genes that had identical direction of changes in both brain structures, a large proportion of genes that had altered expression in one but not in the other region, and only one gene, *Rps17*, that showed opposite expression changes in SN versus striatum. These findings suggest that each brain region has different reactivity to a specific injury that may relate to differences in the type of neurons as well as the neuronal milieu and region-specific microenvironment in the affected areas. As proposed recently, unique combinations of subcellular components including protein translational machinery and ribosomal function may contribute to the selective vulnerability in prion-like disorders including synucleinopathies [81]. In support of this hypothesis the levels of *Rps17*, a ribosomal protein which is part of the protein translation machinery, was differentially altered in two structures with different vulnerability to oligodendroglial α-synucleinopathy and may define or contribute to the early loss of nigral neurons and delayed striatal neurodegeneration. Furthermore, *Rps17* has been associated with modulation of autophagy [82] and cell death mechanisms [83] which in turn may determine the differences in disease events in striatum versus SN. Further detailed studies will be required to precisely identify the role of *Rps17* related to the findings of the current study and their relevance to the different vulnerability to oligodendroglial α-synucleinopathy in striatum and SN.

### miRNAs in MSA–pathogenic and diagnostic role

Several reports have suggested the involvement of altered miRNA expression in the epigenetic landscape of MSA [84]. However, to this end only analysis of end-stage disease post-mortem tissue and of clinically manifested cases has been possible [15;85;86], while it is difficult to assess the participation of miRNA de-regulation in early human MSA. Here, using an MSA transgenic mouse model, we show that such early dysregulation indeed takes place. We identified 59 differentially expressed miRNAs in SN and 33 differentially expressed miRNAs in striatum of MSA mice in a pre-motor disease stage. Among those, miR-433 showed down-regulation in the striatum in the early pre-motor stages of the MSA transgenic model. Interestingly, the expression of miR-433 has been previously reported to be downregulated in the cerebellum of post-mortem MSA cases [85]. Additionally, down-regulation of miR-433 expression has been observed in children with autism [87]. Considering the putative role of miR-433 in striatal neurogenesis [88], the current findings in the transgenic MSA model and its relevance to the findings in the human disease strengthen the possible role of this specific candidate in the early stages of MSA neurodegeneration. Furthermore, miR-433 is involved in the regulation of HDAC6 expression [89;90] that has been shown to co-localize with α-synuclein in GCIs in the MSA brain [91;92]. The current gene expression analysis showed unchanged *HDAC6* gene expression in MSA mice in the early pre-motor stage of disease. Taken together these data suggest that miR-433 expression changes in the early pre-motor stages of MSA may precede alterations of *HDAC6* expression that may play an important role in the pathogenesis of the disease in its later stages.

MicroRNA dysregulation has been previously assessed in a transgenic MSA model with full onset of the pathology and in human post-mortem MSA brains [15]. Thereby, expression of miR-96 was found to be up-regulated in the later stages of disease in both the mouse model and human MSA, respectively, but our screening did not confirm significant dysregulation of miR-96 expression in striatum and SN in the early pre-symptomatic MSA model. This discrepancy may be related to changes in the miRNA profile with the progression of the disease that may reflect different events in the early and late stages of MSA.

By contrast, we found miR-19b to be significantly down-regulated in both striatum and SN in the pre-motor stage of disease in MSA mice and although the predictive value of the disease stages in the transgenic models and their relevance to human MSA may seem uncertain, a very strong support of our finding comes from a recent study by Fernandez-Santiago and co-workers [93] who identified for the first time that miR-19b down-regulation occurs in prodromal (preceding motor or cognitive symptomatology) stages of synucleinopathies. Conversely, the MSA model of established pathology described by Ubhi et al. showed significant up-regulation of miR-19b similar to end-stage MSA [15]. Taken together, these findings show that miRNA expression is variable throughout the course of the disease and that downregulation of miR-19b is a strong candidate for an early MSA marker as proposed already in a clinical trial for PD and DLB [93], while its upregulation appears to be associated with advanced disease progression.

### General miRNA-mRNA regulatory network in MSA: towards new targets for disease modification

Direct overexpression of the α-synuclein gene in oligodendroglia of the MSA transgenic mice has been shown to trigger early oligodendroglial dysfunction related to disrupted myelination and changes in oligodendroglial differentiation relevant to the human disease [62;63]. It is interesting to note, however, that with the stringent correlation analysis of putative miRNA-mRNA interactions, we did not obtain evidence for a significant involvement of miRNAs on the modulation of genes linked to oligodendroglial dysfunction. However, at this stage and with the applied methodology it is not possible to exclude that cell-specific (i.e. neuronal versus glial) alterations associated with miRNA-mRNA interactions occur in the MSA brain.

By contrast, our data strongly suggest an involvement of a miRNA-mRNA regulatory network in the control of early inflammatory responses mainly linked to dysregulated phagocytic activity of macrophages that may be associated with an early deficit in the clearance of α-synuclein as previously suggested [23;94]. Previous neuropathological, imaging and experimental studies have confirmed the contribution of microglial activation to the MSA disease process [56;57;95], but the understanding of its progression and participation in the various stages of the disease is yet unclear. Due to the lacking insights into the mechanisms of neuroinflammatory control in MSA, most of the therapeutic strategies targeting neuroinflammation in MSA (similar to other neurodegenerative disorders) to date fail to exert functional benefit in spite of the successful suppression of microglial activation [96;97]. It will be interesting to see in the future, if the predicted involvement of miRNA regulation can be confirmed, which would open the field to pursue new candidate treatment targets to support the clearance of α-synuclein in MSA through microglia.

Further putative miRNA-mRNA networks were predicted by our data set in relation to processes such as protein handling, transmembrane transport and cell death. Some of these processes have previously been linked to human MSA thus encouraging further elucidation. For instance, involvement of HSP70 and HSP90 in the formation of GCIs was suggested by earlier neuropathological studies [98;99], and it will be relevant to identify to what extent putative miRNA-mRNA interactions linked to the expression of *Hspa5* and *Hsp90ab1*, respectively, may direct the disease process and α-synuclein processing in MSA. We also identified several miRNA-mRNA candidate networks that may contribute to the neurodegeneration in the MSA mouse brain but have not been linked to this process before (e.g. controlling calcium transport in the striatum (*Cacnb4*, *Ryr2*)). These might be interesting targets for future neuropathological studies.

### Limitations, clinical relevance and conclusions

In summary, our study provides first insights into the early regulatory miRNA-mRNA network during the pre-motor stage of MSA as studied in a transgenic mouse model with oligodendroglial α-synuclein accumulation and progressive SND, featuring the Parkinsonian variant of MSA (MSA-P). The etiopathogenesis of human MSA is poorly understood to date, and it is unclear what triggers α-synuclein accumulation in oligodendrocytes of human MSA or how long before the clinical onset this process occurs. One apparent limitation of the currently used mouse model of MSA is the fact that the transgenic MSA mouse replicates GCI pathology by forced overexpression of *SNCA* in oligodendroglia. Current prion-like propagation studies of α-synuclein claim MSA to be the next prion disease [100;101]. However, none of these propagation studies has been able to generate wide-spread GCI pathology, suggesting that the mechanism of prion-like spreading may be relavent to MSA pathogenesis/progression at a later stage of the disease, but needs a specific yet unknown trigger for α-synuclein accumulation in oligodendroglia. Changed expression of *SNCA* in MSA oligodendrocytes as recently suggested [7] may provide such a trigger. Therefore, even though the trigger for GCI formation is obviously different between the human disease and the mouse model, the downstream events may very well be similar rendering our findings relevant to the understanding of the human disease mechanism. A limitation of our analysis is the fact that the identified changes in mRNA and miRNA expression in SN and striatum cannot be attributed to a specific cell type in the affected regions with the current methodological approach, but may give directions for further studies. The current experiments give directions towards the major biological processes and functions that may be under miRNA-mRNA network conrol in the early MSA pathogenesis, however further in-depth functional studies will need to address selected miRNA/mRNA candidates to specifically define their role in the disease process. Based on the current screening we confirm the role of changes in the immunological/inflammatory responses as well as oxidative stress even in the very early pre-motor stages of MSA. Other mechanisms related to human MSA like oligodendroglial dysfunction, lipid metabolism, and protein handling were confimed in the PLP-α-synuclein mouse model and point towards early transcriptome de-regulation that may trigger MSA deficits and pathology in the early stages of the disease. Unravelling the early events in the pathogenic cascade of MSA may provide an important step toward identifying early disease markers in at risk cohorts of patients and candidate therapeutic targets for this devastating disease.

## Supporting Information

S1 Fig (A) Overview of the experimental set-up. (B) Overlap of differentially expressed mRNAs resulting from microarray and RNA-seq analysis in SN. (C) To provide verification of the expression analysis defined by the two different methods we performed correlation analysis as demonstrated in a bladaltmanplot of the differences plotted against the averages of the two measurements (mRNA measured with RNA-seq (DESeqFC) and mRNA measured with microarray (ChipFC)). Horizontal lines are drawn at the mean difference, and at the limits of agreement, which are defined as the mean difference plus and minus 1.2 times the standard deviation of the differences, indicating that on an average the agreement between the two techniques is quite good. X-axis: mean of the both techniques; Y-axis: difference between the 2 values. (D) Heatmap shows mean fold changes (log_2_ transformed) of differentially expressed mRNAs in SN as identified by microarray and RNA-seq analysis. The color gradient shows positive and negative log_2_ transformed fold changes in orange and blue color, respectively. (E) KEGG pathway analysis of deregulated mRNAs in striatum of MSA mice in a pre-motor stage of the disease. (F) KEGG pathway analysis of deregulated mRNAs in SN of MSA mice in a pre-motor stage of the disease. Color code represents the significance (p-value) of the KEGG pathway enrichment. Length of the bars corresponds to the number of differentially expressed genes linked to each pathway.
(JPG)

S2 Fig Up-regulated miRNAs (right) and down-regulated miRNAs (left) in SN of MSA mice with the number of their predicted deregulated targets (expressed by blue color gradient). Predicted targets were assigned to enriched GO-terms (indicated at the bottom).
(JPG)

S3 Fig Up-regulated miRNAs (right) and down-regulated miRNAs (left) in striatum of MSA mice with the number of their predicted deregulated targets (expressed by blue color gradient). Predicted targets were assigned to enriched GO-terms.
(JPG)

S4 Fig Real-time PCR verification of RNA-seq and microarray data for representative mRNAs in striatum and substantia nigra (SN). (JPG)

S5 Fig Real-time PCR validation of the data for representative mRNA-miRNA predicted pairs in the striatum. (JPG)

S1 Table List of primers used for RT-PCR analysis. (DOCX)

S2 Table List of differentially expressed mRNAs in the striatum of MSA mice in a pre-motor stage of the disease. (XLSX)

S3 Table List of differentially expressed mRNAs in the SN of MSA mice in a pre-motor stage of the disease. (XLSX)

S4 Table List of enriched GO terms linked to differentially expressed mRNAs in the striatum of MSA mice in a pre-motor stage of the disease. (XLSX)

S5 Table Clusters of GO biological processes and corresponding deregulated mRNAs in striatum relevant to human MSA defined by REVIGO analysis. (XLSX)

S6 Table List of enriched GO terms linked to differentially expressed mRNAs in the SN of MSA mice in a pre-motor stage of the disease. (XLSX)

S7 Table Clusters of GO biological processes and corresponding deregulated mRNAs in substantia nigra relevant to human MSA defined by REVIGO analysis. (XLSX)

S8 Table List of differentially expressed miRNAs in the striatum of MSA mice in a pre-motor stage of the disease. (XLSX)

S9 Table List of differentially expressed miRNAs in the SN of MSA mice in a pre-motor stage of the disease. (XLSX)

S10 Table List of miRNAs containing information about their predicted target genes and correlation values. This list was filtered for miRNA-target interactions, which (i) were predicted by mirWalk 2.0 with a p-value < 0.1, or (ii) were predicted by at least two prediction programs, or (iii) feature a validated target gene. In all cases, a minimum correlation value of -0.3 was required.
(XLSX)
