# Supplementary figures and images for: Changes in the miRNA-mRNA Regulatory Network Precede Motor Symptoms in a Mouse Model of Multiple System Atrophy: Clinical Implications
Source: PLoS One. 2016 Mar 10;11(3):e0150705. doi: 10.1371/journal.pone.0150705 (PMC4786272; doi:10.1371/journal.pone.0150705)

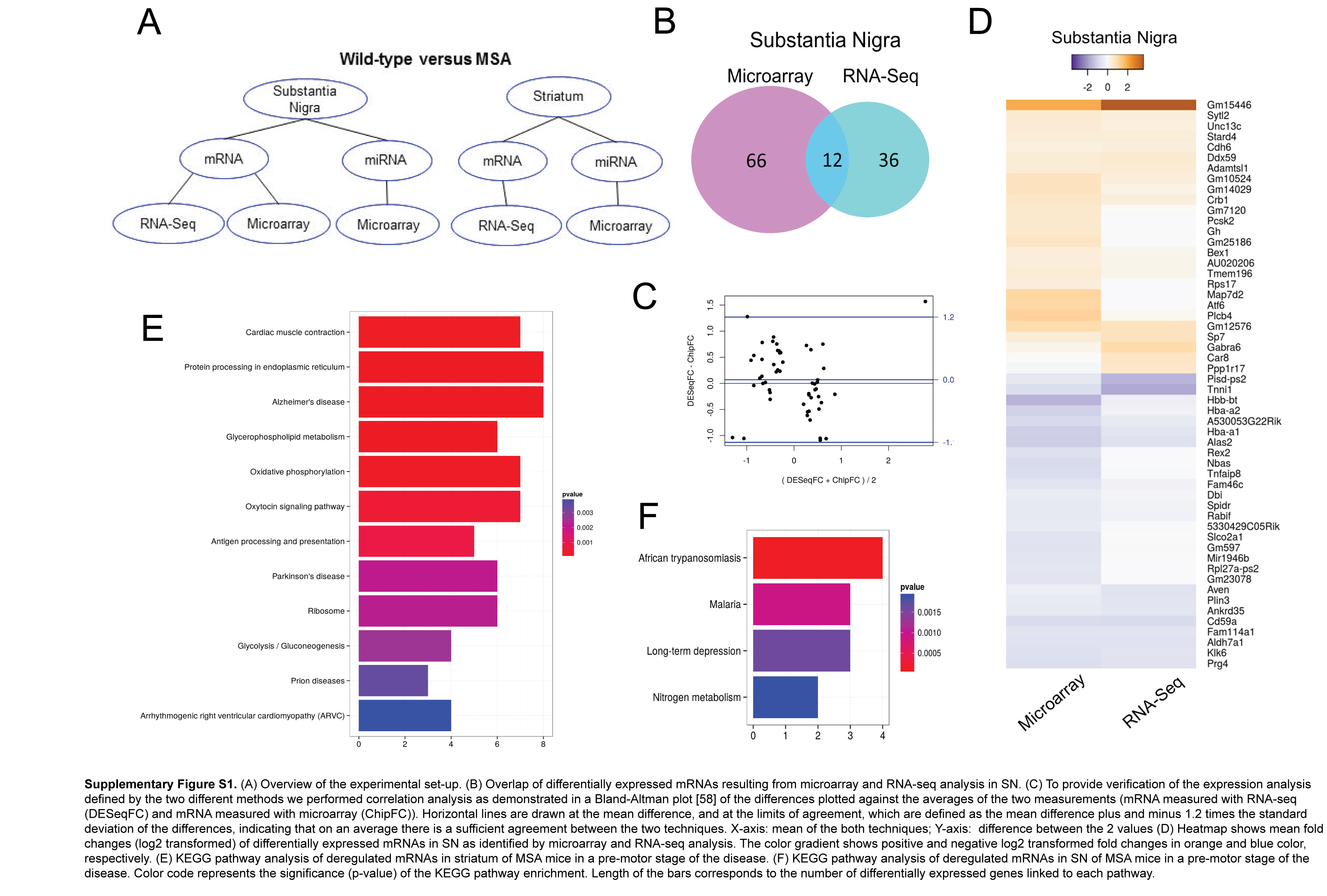

Supplement: S1 Fig — (A) Overview of the experimental set-up. (B) Overlap of differentially expressed mRNAs resulting from microarray and RNA-seq analysis in SN. (C) To provide verification of the expression analysis defined by the two different methods we performed correlation analysis as demonstrated in a bladaltmanplot of the differences plotted against the averages of the two measurements (mRNA measured with RNA-seq (DESeqFC) and mRNA measured with microarray (ChipFC)). Horizontal lines are drawn at the mean difference, and at the limits of agreement, which are defined as the mean difference plus and minus 1.2 times the standard deviation of the differences, indicating that on an average the agreement between the two techniques is quite good. X-axis: mean of the both techniques; Y-axis: difference between the 2 values. (D) Heatmap shows mean fold changes (log2 transformed) of differentially expressed mRNAs in SN as identified by microarray and RNA-seq analysis. The color gradient shows positive and negative log2 transformed fold changes in orange and blue color, respectively. (E) KEGG pathway analysis of deregulated mRNAs in striatum of MSA mice in a pre-motor stage of the disease. (F) KEGG pathway analysis of deregulated mRNAs in SN of MSA mice in a pre-motor stage of the disease. Color code represents the significance (p-value) of the KEGG pathway enrichment. Length of the bars corresponds to the number of differentially expressed genes linked to each pathway. (JPG) [file pone.0150705.s001.jpg]

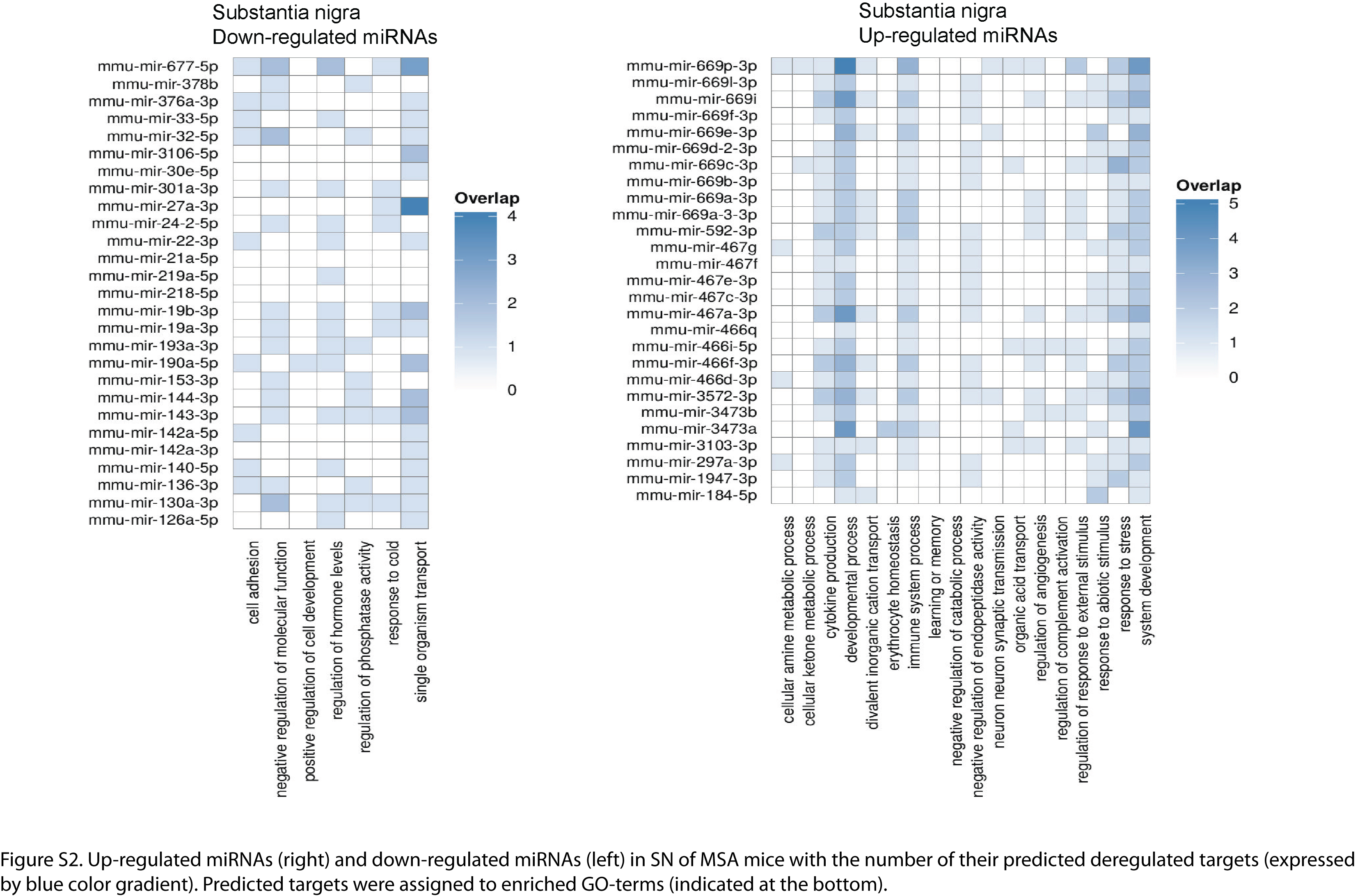

Supplement: S2 Fig — Predicted targets were assigned to enriched GO-terms (indicated at the bottom). (JPG) [file pone.0150705.s002.jpg]

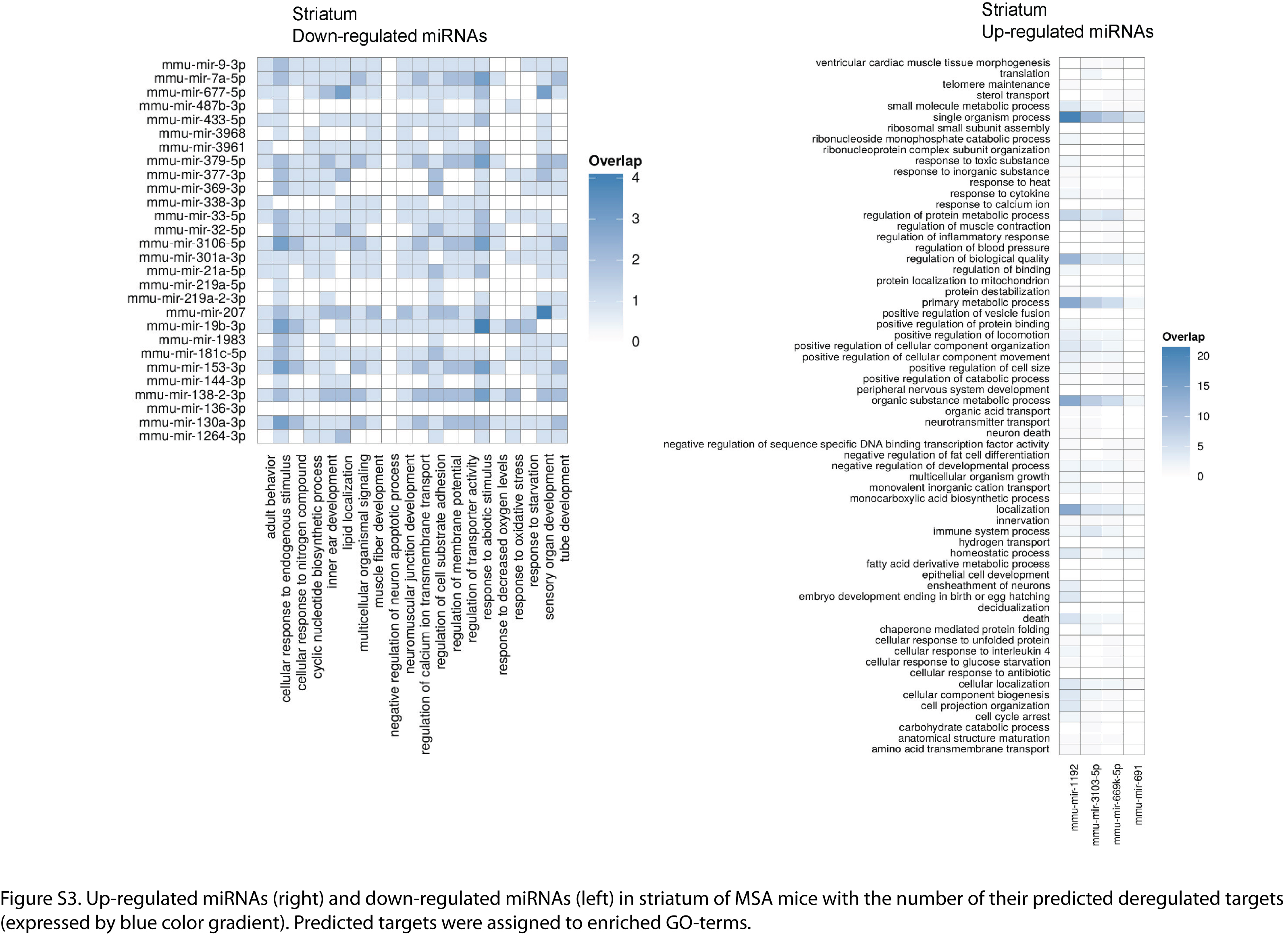

Supplement: S3 Fig — Predicted targets were assigned to enriched GO-terms. (JPG) [file pone.0150705.s003.jpg]

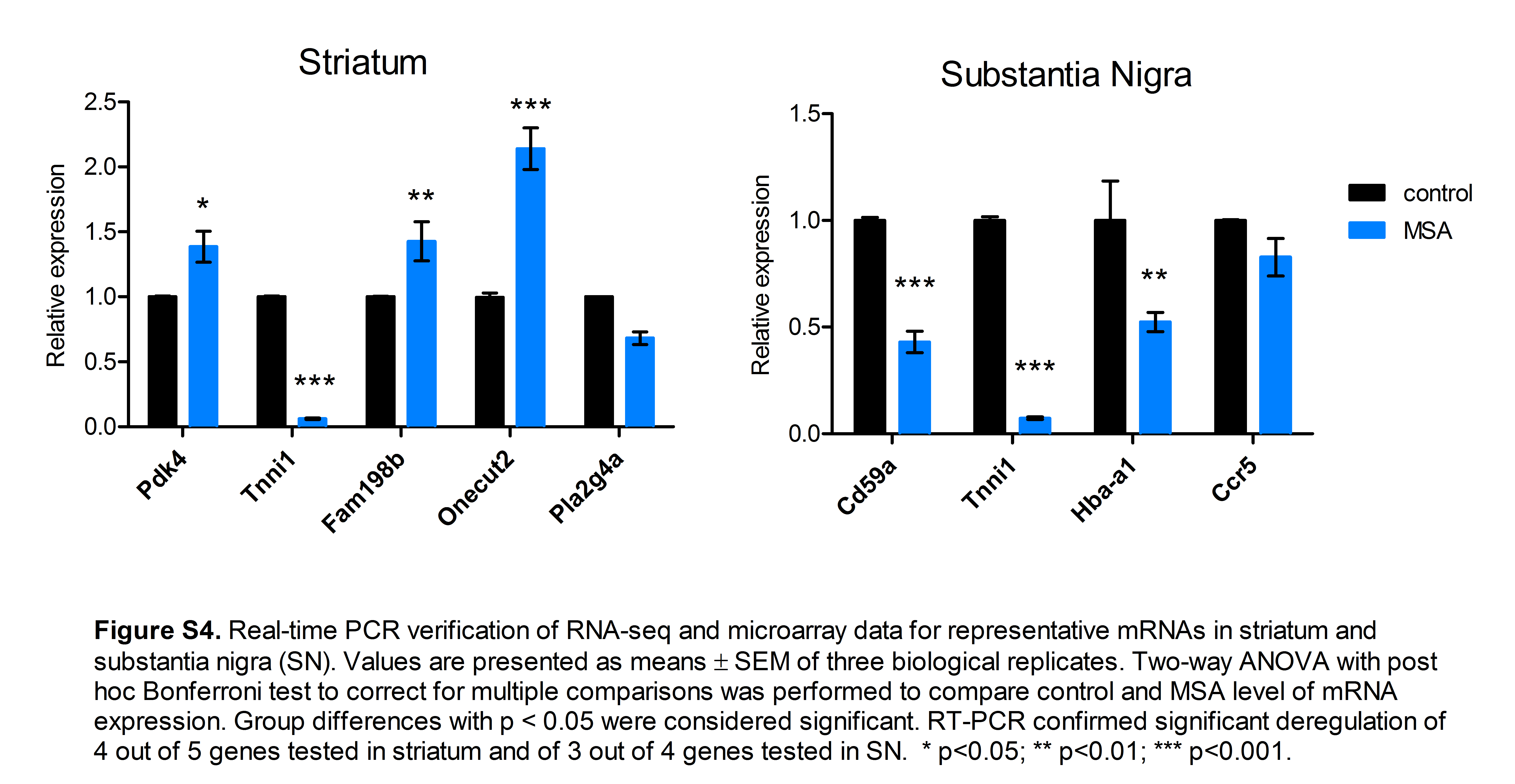

Supplement: S4 Fig — (JPG) [file pone.0150705.s004.jpg]

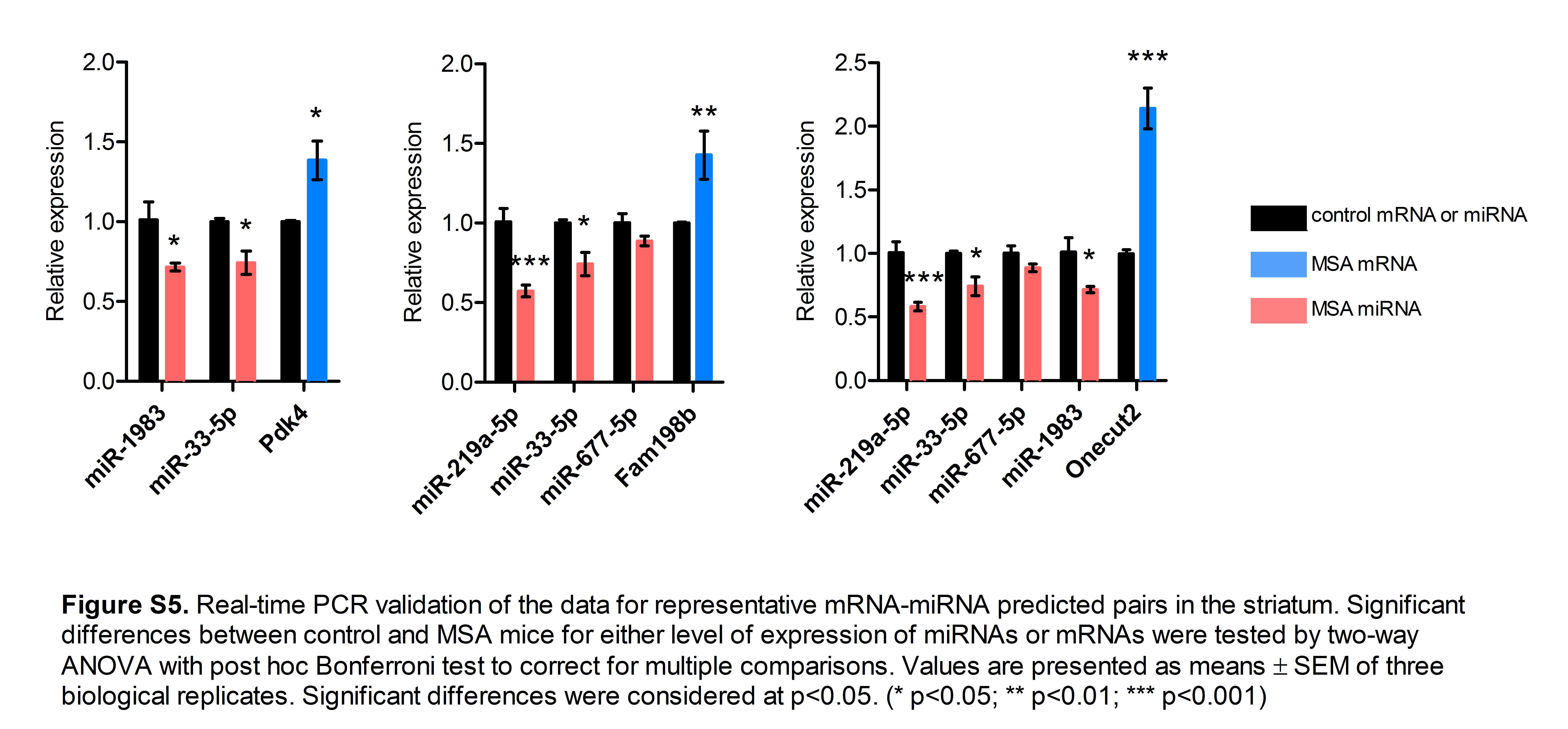

Supplement: S5 Fig — (JPG) [file pone.0150705.s005.jpg]
