# Supplementary material for: Changes in the miRNA-mRNA Regulatory Network Precede Motor Symptoms in a Mouse Model of Multiple System Atrophy: Clinical Implications
Source: PLoS One. 2016 Mar 10;11(3):e0150705. doi: 10.1371/journal.pone.0150705 (PMC4786272; doi:10.1371/journal.pone.0150705)
Supplement: S1 Table — (DOCX) [file pone.0150705.s006.docx]

| **Transcript name** | **Forward primer (5’-3’)** | **Reverse primer (5’-3’)** |
| --- | --- | --- |
| **mRNA** | | |
| Pdk4 | AGTGACTCAAAGACGGGAAAC | GTGTGAGGTTTAATTCTGGCG |
| Onecut2 | AGTAAACTCAAATCTGGCAGGG | TGTTTGGTTCTTGCTCTTTGC |
| Fam198b | TGGACATGAGTGAAGTGTTCG | TGCTAGAGATGAGTCCCAGAG |
| Pla2g4a | ACCTGCCATATCCCTTGATTC | GTTCATTTTCGCCCACTTCTC |
| Cd59a | GCCTCACATGCTACCACTG | CCAACACCTTTGATACACTTGC |
| Tnni1 | TCTAAGCACAAGGTGTCCATG | CCAGACATAGCCTCCACATTC |
| Hba-a1 | ATCCCGTCAACTTCAAGCTC | CTTAACGGTACTTGGAGGTCAG |
| Ccr5 | TCCAGCAAGACAATCCTGATC | AACCATTCCTACTCCCAAGC |
| **miRNA** | | |
| miR-219a-5p | TGATTGTCCAAACGCAATTCT | miScript universal primer (Qiagen) |
| miR-677-5p | TTCAGTGATGATTAGCTTCTGA | miScript universal primer (Qiagen) |
| miR-1983 | CTCACCTGGAGCATGTTTTCT | miScript universal primer (Qiagen) |
| miR-33-5p | GTGCATTGTAGTTGCATTGCA | miScript universal primer (Qiagen) |
| U6 | CGCTTCGGCAGCACATATAC | miScript universal primer (Qiagen) |

**Supplementary Table S1.** List of primers used for RT-PCR analysis.
